# Social Embeddedness of Firefighters, Paramedics, Specialized Nurses, Police Officers, and Military Personnel: Systematic Review in Relation to the Risk of Traumatization

**DOI:** 10.3389/fpsyt.2020.496663

**Published:** 2020-12-21

**Authors:** Renate Geuzinge, Merel Visse, Joachim Duyndam, Eric Vermetten

**Affiliations:** ^1^Humanism and Social Resilience, University of Humanistic Studies, Utrecht, Netherlands; ^2^Care Ethics, University of Humanistic Studies, Utrecht, Netherlands; ^3^Department of Psychiatry, Leiden University Medical Center, Leiden, Netherlands; ^4^Ministry of Defense, Military Mental Health Research Center, Utrecht, Netherlands; ^5^ARQ National Psychotrauma Center, Diemen, Netherlands

**Keywords:** trauma, high-risk professionals, social embeddedness, environment, relationships

## Abstract

**Background:** Firefighters, paramedics, specialized nurses working in Intensive Care Units (ICUs), Operating Rooms (OR), and Emergency Rooms (ER), police officers and military personnel are more frequently exposed to potentially traumatic events than the general population; they are considered high-risk professionals. To reduce the risk of traumatization it is of great importance to be embedded in a social environment with supportive relationships.

**Methods:** We performed a systematic review (based on the PRISMA-Guidelines) looking for social connections within the environment in which high-risk professionals are embedded (work, home, community), to obtain evidence on the impact of these connections on the risk of traumatization. Additionally, we aim to identify relevant supportive relationships in the professionals' environments. We identified the relevant scientific literature by searching, without time, and language restriction, five electronic bibliographic databases: MEDLINE, PsycINFO, Sociological Abstracts, CINAHL, and Web of Science. These databases were last searched in January 2019.

**Results:** A qualitative analysis of the 89 eligible (out of 9,047 screened) studies shows that for firefighters, paramedics, and emergency nurses social connections in their work environment are predominantly supportive relationships and may protect them against traumatization. In other occupations (OR-nurses, ICU-nurses, police officers), however, social connections at work are not only a source of support but are also a source of stress. For military personnel study results are inconclusive as to whether their social connections at work or at home support them against traumatization. In so far as connections are supportive, their sources vary greatly from one occupational group to another; they differ between work vs. home as well as within work between peers vs. supervisor.

**Conclusions:** Being embedded in a social environment, i.e., having social connections, is important but not always sufficient to protect high-risk professionals against traumatization. For, while these connections may be the antecedents of supportive relationships, they can also be the antecedents of damaging relationships. Additionally, the sources of supportive relationships differ among groups. This suggests that knowledge of how the social structures of the occupational groups differ may increase our understanding of the impact of social connections and relationships, including socialization, on the risk of traumatization of high-risk professionals.

## Introduction

Firefighters, ambulance paramedics, police officers, and military personnel are more frequently exposed to potentially traumatic events than the general population (1–3). Their involvement in critical incidents, which are often a matter of life and death of others and sometimes of their own, may go along with high arousal or dissociative states and feelings of powerlessness. They risk the development of posttraumatic stress disorder (PTSD) (1, 4–6), or other mental health problems related (comorbidity) to traumatization (7, 8). Exposure to human suffering and indirect exposure to extreme stressful events can also result in secondary traumatic stress disorder, compassion fatigue, or burnout (9–11). Any lower level of mental health can compromise the mental capacity to integrate stressful events and thus increases the risk of traumatization (12, 13). That is why they are considered to be a high-risk category. Exposure to critical and potentially traumatic events not only concerns the aforementioned occupations, but also professionals working intramurally. Emergency and operating room nurses are also exposed to the severely injured patient. Although gruesome wounds are often physically covered by the time a patient arrives at the intensive care unit, the nurses are still exposed to the underlying stories of pain and suffering. Reviews confirm that specialized nurses working in intensive care units, emergency, and operating rooms also report significant mental health problems (14–24). It would be incorrect to assume however, that exposure to these events inevitably leads to dysfunctional or pathological reactions (25). Most high-risk professionals are not affected by traumatic stress and stay on the job (26), and numerous studies even report positive and salutary outcomes as a result of the experience of occupational trauma, i.e., posttraumatic growth (27–29). What may account for these different outcomes?

High-risk professionals were “all too often expected to be invulnerable to emotions” [(30), p. 4] in the presence of the distress of others, and therefore sometimes viewed as super-human with exceptional emotional strength or even, by the general population, as “heroes” (31, 32). Meta-analytic reviews of studies conducted across a wide range of general adult populations did indeed reveal several clear predictive personal variables with respect to PTSD risk, such as pretraumatic experiences, peritraumatic distress or peritraumatic dissociation, and the experience of subsequent stressful life events (5, 33).

Expectations about special, personal qualities to overcome stressful events may be the result of early research efforts that focused on “resilient children” (34–36). This changed when it became clear that resilience often derives from environmental resources. This can be seen as a reflection of the established structure-agency debate in sociology in that early researchers of development and resilience were later criticized of having been too agency centered, while ignoring any “structural forces” (37). We wonder whether this neglect of the social environment might still be present in studies on high-risk professionals. When resilience is taken as a personal trait (38) we may inadvertently pave the way for perceptions that some individuals simply do not “have what it takes” (34, 35). This is an individual-focused perspective that can easily lead to blaming the person (38).

### Conceptual Clarification

When focusing on available environmental resources in adult populations, meta-analytic reviews found social support to be the single most protective factor (5, 33). Significant others can protect people who deal with stressful and potential traumatic events against traumatization. Their responding and offering of support may diminish the impact of these events. The perception that one can rely on the social connections in the environment to provide the necessary support may redefine a difficult situation as being less threatening, as well as enhance a person's sense of control, self-esteem, optimism and reinforce one's coping efforts, and/or regulation of emotions (39–43). Although at first glance the construct social support may seem to measure an environmental resource, the way social support is conceptualized (40, 44, 45) reveals that personal variables play a large role as well.

Next to this, we noticed that different high-risk occupations are often lumped together in one study, thereby ignoring differences in the environment of the distinct professions. Both concepts will be clarified underneath and will restrict the focus of this review.

### Social Support: Offered vs. Perceived

Social-emotional support is a multidimensional concept. It is commonly conceptualized by three constructs; social embeddedness, enacted social support, and perceived social support (46, 47). Social embeddedness refers to the connections that individuals have with others in their social environment. Being socially connected is a central element in one's psychological sense of community. This sense of community (48–50) is associated with a wide range of positive psychological outcomes (51–53). It generally refers to a geographically defined group and to the ties that link people to their place of residence. Some researchers also applied this concept to organizations and work settings (54–57) and a few specifically to high-risk professionals (58). A function of social embeddedness is enacted support, which is conceptualized as actions that significant others perform when they offer assistance, i.e., actual social support (40, 46, 59) or supportive relationships. These naturally occuring supportive relationships are not the same as a top-down policy driven procedure such as professional support or formal peer support, which is therefore not taken into account.

Perceived social support refers to how individuals perceive and appraise enacted or actual social support (60). It is, crucially for this review, not an environmental factor as it is influenced by how the relationships, through which it is offered, are perceived (60). When two or more people interact in support, there are by default two perspectives involved: providing and receiving support (45, 46, 61). Whether or not people accept or make use of actually offered support depends upon their personal capacity to perceive it.

### Different Occupations

The literature on high-risk professionals consists primarily of studies of comparable but undistinguished occupational groups, and researchers have made few attempts to distinguish subgroups within this broad category. Firefighters, police officers, emergency service, and ambulance personnel are often lumped together in one single category such as “first responders” (64, 65) or “critical occupations” (30).

The background may be that some highly influential researches, among which those of Beaton and Murphy (66) and Beaton et al. (10), took place in the United States (U.S.). In the U.S. firefighters are also trained as Emergency Medical Technicians (EMTs) or some as paramedics. As EMTs they are also called upon to render emergency medical treatments, such as cardiac arrests and other first medical aid; in one study (66) a firefighter/EMT group reported that over 60% of their “runs” were of such an emergency medical nature. In some rural (remote) areas of the U.S. firefighters are even trained as paramedics, which increased the percentage to over 80% (66). Not surprisingly, researchers found comparable outcomes among firefighters and paramedics concerning operational stressors, such as gruesome victim incidents (10), and organizational stressors, such as conflict with administration (66). Therefor they concluded that their occupational duties as well as their training and work “cultures” are comparable. To use these outcomes to group these two occupations together, however, does not seem justified as not all paramedics are also firefighters nor are all firefighter also paramedics.

We do not take into account professionals who experience incidents infrequently. For, while dealing with natural disasters and terrorist attacks, individuals and organizations may temporarily replace their traditional social structures and functions with temporary ones so that social relationships in the environment may change temporarily too (62, 63). To summarize, high-risk professionals cannot be taken as one single category, as specific environmental factors have to be taken into account. Additionally, if we intend to explore the influence of the social environment on traumatization of high-risk professionals, we should exclude studies that solely measured perceived social support.

### Objectives

This systematic literature review aims to enhance insight into the social connections within the environment in which high-risk professionals are embedded (work, home, community), and the influence of these connections on the risk of traumatization. Additionally, we aim to identify supportive relationships in the professionals' environments.

## Methods

### Selection Criteria

Included in this review are published studies that focused on the mental health of professionals in relation to social connections or relationships at home, in their communities or their work in military, police, ambulance, and fire departments, as well as in intensive care units, operating rooms, and emergency rooms. While mainly focusing on studies about traumatization we decided to broaden our search to studies that included other mental health constructs.

Excluded from this review are studies in which samples of different occupations are grouped together, e.g., medical specialists with specialized nurses, or in which the occupational group was not specified by the researchers. Studies on professionals involved in rescue and disaster operations, and studies that focused on the effect of formal social support (e.g., debriefing or mental health treatment) are also excluded from this review. Generally, the following exclusion criteria are also applied: discussion and opinion papers; studies available merely in the form of abstracts or presentations; duplicates or papers not written in the Latin alphabet.

To improve the likelihood of identifying all relevant papers we decided not to restrict this systematic review to quantitative research only. Qualitative research may be important in identifying the role of social connections or relationships in the environment as well, as it may provide important information about phenomena such as the feeling of belonging, emotional connection, or qualitative aspects of relationships.

This review was conducted in line with the Preferred Reporting Items for Systematic Reviews and Meta-Analyses (PRISMA) guidelines (67) (see Supplementary Material 1). However, this review has not been subjected to meta-analysis due to the heterogeneity of the constructs and their assessments.

### Search Strategy

The research group identified the relevant scientific literature by searching, without time and without language restriction, five electronic bibliographic databases including: MEDLINE, PsycINFO, Sociological Abstracts, CINAHL, and the citation indexing service of Web of Science. These databases were last searched in January 2019.

Within each professional domain (P), papers were searched for containing at least one term or synonym from the block “mental health” (O). In the MEDLINE, PsycINFO, and Sociological Abstracts databases was the search narrowed for some professional domains (police officers and military personnel), by adding the block “social environment” (I). Major terms and their synonyms, or related terms, were derived from keywords of papers during a pilot search (see Supplementary Material 2 Major Search Terms). For an example of the used search strings for all professional domains in two of the databases, see Supplementary Material 3. (An outline of the full used search terms and search strings for all occupational groups and all five databases is available on request).

### Study Selection

**Figure 1:**
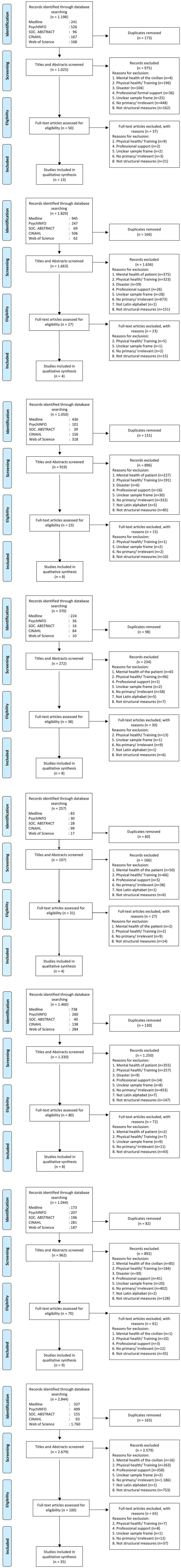
PRISMA-based Flow-Charts of each occupational group. From Moher et al. (67).

The flow charts documenting the selection process for each professional group can be viewed in Figure 1. After duplicates were removed, titles and abstracts were screened for this first selection process by two reviewers (RG and MV). Disagreements between them were resolved by a third reviewer (EV). In the second selection process full texts of all potential relevant studies were read by RG, MV, and EV. Doubts between these authors regarding article eligibility, or key criteria during extraction were resolved through discussion with the remaining author (JD).

During the second selection process we decided to sharpen the selection by excluding all studies which did not measure or address the outcome of any mental health constructs of the professional but focused on job satisfaction or commitment toward the organization's mission, “end-product” or service. Though job satisfaction might be expected to correlate positively with, for example, a sense of community in the workplace, job satisfaction is not a necessary, nor a sufficient condition for the experience of a sense of belonging or community at work (56).

### Data Extraction and Synthesis

For each included study we extracted the following data: authors, year of publication, country, journal, study design, professional group (sample characteristics, context), measured constructs and measurements. Risk of bias (RoB) of individual studies is assessed with a tool for cross-sectional studies and surveys (68). For a full list of eligible articles of each professional group, see Supplementary Table 1.

To give an overall impression of the studies included in this review, eligible articles for each professional group are summarized in the following results section.

## Results

In total, 89 studies were eligible for inclusion in this review. Except seven, all were published after 2000. This section first provides an overview of the results from studies that focused on occupations in emergency services organizations starting with firefighters and ambulance paramedics, to be followed by specialist areas of nursing. Nurses in emergency rooms, operating rooms and intensive care units are discussed separately. Next, the results from studies that focused on armed services organizations are discussed, namely police officers and military personnel. Every section of an occupational group begins with a summary of quantitative findings.

In synthesizing the qualitative results per group, we first summarize studies on general embeddedness, i.e., the connections to potential supportive others in the professionals' social environment (at work, at home, or in their communities, respectively). Under this heading we also include outcomes on a sense of community and organizational belongingness, the last refers to the extent employees feel valued and respected by their organizations (69) or administrators. This section is followed by studies that focused on relevant supportive relationships in which the professional is embedded; at work with peers and supervisors, to be followed at home with family members and friends.

### Firefighters

#### Study Characteristics

The search identified 196 articles on the mental health of firefighters, of which 13 addressed social connections in the environment (7%). All studies were quantitative, of which two included a longitudinal design. Eleven studies focused on general embeddedness under the following constructs: a sense of organizational membership, belongingness, social bonding and connection, camaraderie, organizational connectedness or disconnectedness, harassment, and workplace discrimination. Four studies (also) focused on relevant supportive relationships within the organization, i.e., supervisors/coworkers (70), relationships between employees, supervisory relations; two of these also expanded their focus to the relationships of the professional outside the fire department, i.e., family and friends.

#### General Embeddedness

Professional firefighters engage not only in work-related tasks but also live together for extended periods of time, usually 3-day, 24-h shifts (71, 72). A study among U.S. firefighters in six different firehouses investigating quality of life and social bonding showed that those with a low level of social bonding and connection, although low prevalent, are also more likely to report poor mental health. The instrument developed for this study was not subjected to psychometric testing. Nevertheless, it allowed the researchers to assess how frequently these firefighters engaged in activities related to social bonding. Out of these 112 firefighters 96% had a high score on these activities (73). One study (74) found that firefighters' psychological sense of (work-) community was positively impacted by their satisfaction with social support from other coworkers. Firefighters that were satisfied with the support experienced less stress with providing care to victims than those who experience low levels of support satisfaction. A lower report of thwarted belongingness (cf. disconnectedness) was associated with lower levels of PTSD symptoms (75, 76). In a small subgroup sample of wildland firefighters this feeling of disconnectedness was associated with higher suicide risk (76).

A cross-sectional study among Australian volunteer firefighters found that camaraderie—defined as a feeling of belonging, a sense of shared identity, reciprocal trust, and the strong positive bonds that exist within cohesive work groups—is a protective factor against posttraumatic stress (77). However, in a study among professional Australian firefighters organizational belongingness was not a predictor of reduction in PTSD symptoms (78) but might play a mediating role in the development of Post Traumatic Growth (PTG) (79).

Results from a study among Finnish firefighters indicated that positive interaction with coworkers may have long-term effects on work engagement and self-esteem, and consequently on (future) work ability (80).

Korean firefighters who experienced workplace discrimination had a higher likelihood of depressive symptoms (81). Among female U.S. firefighters who reported having a history of (sexual) harassment while on the job, significant higher levels of PTSD symptoms were found (82).

#### Relevant Supportive Relationships

##### Supervisors

The study among Finnish firefighters found the same results for support and positive feedback from one's supervisor (80). Spanish firefighters' emotional social support from supervisors and coworkers is positively related with the ability to face up to adversities in life (resilience), but actions from people in leadership positions denoting support, recognition, and social companionship are especially important to these firefighters (83).

##### Home

Huynh et al. (84) showed through a longitudinal design that support from family and friends not only protects Australian volunteer firefighters from burnout (cynicsm), but it also helps them to stay connected to volunteering.

### Paramedics

#### Study Characteristics

Of all the identified articles on the mental health of ambulance paramedics (170), four addressed social connections in the environment (2%). All studies were quantitative studies, of which one included a longitudinal design. Two studies focused on general embeddedness, i.e., workplace belongingness and connectedness. One study focused specifically on potential supportive relationships outside the organization, i.e., with a spouse. Two studies (also) focused on relevant supportive relationships, i.e., peer and supervisor.

#### General Embeddedness

Workplace belongingness (i.e., a sense of organizational membership) showed to have impact on the mental health of Australian ambulance officers who experienced a traumatic event. It reduced their distress levels and enhances their ability to bounce back or recover from stress (85).

Another study amongst Canadian couples, of which one is a paramedic, investigated the social environment at home (marital interaction and coping strategies) in relation to occupational stress and burnout (emotional exhaustion and depersonalization). Significant associations were observed between paramedics' work stress, the subsequent increased rumination and interpersonal withdrawal at home. Not only the paramedics withdrew from interactions but their spouses too, which can be considered an impairment of the social resource (potential support) for the paramedic (86).

#### Relevant Supportive Relationships

##### Peers

Lewig et al. (69) used a sample of 487 rural volunteer ambulance officers in Australia to develop a better understanding of the relationship between connectedness, and mental health problems and turnover. Results showed that when volunteers feel supported by their peers they were less likely to become overly exhausted or cynical about their volunteering obligations and more likely to continue.

##### Supervisors

A large sample size study in Australia demonstrated the important role of support from supervisors on symptoms of common mental disorder and psychological well-being of ambulance personnel (87).

### Emergency Room Nurses

#### Study Characteristics

The search identified 103 articles on the mental health of emergency room nurses of which eight addressed social connections in the environment (8%). All eight studies were quantitative and included a cross-sectional design, of which one longitudinal. Three of these studies focused on general embeddedness i.e., feeling supported by the hospital administration after potentially traumatic incidents or the relations with physicians. Six studies (also) focused on relevant supportive relationships within the organization i.e., supervisors and colleagues and one study included the social relationships outside the organization (i.e., family, peers and religious groups).

#### General Embeddedness

Two small sample size studies conducted in Ireland and Canada examined the effects of stress-related incidents in the emergency department (88, 89). The Canadian study found that interpersonal conflict with coworkers is significantly associated with PTSD symptoms (89). Both studies found that the majority of respondents reported they had received no (74%) or inadequate (20%) support from their hospital employer to help them deal with acute stress (88) or traumatic incidents (67%) (89). Considering the relatively small samples of these two studies (90 and 51), and their unclear sampling frames (as other professionals working in the emergency department were included) we should refrain from drawing specific conclusions about emergency room nurses.

Similar to other specialized nurses (90) emergency room nurses collaborate closely with physicians. In a small sample size study among 64 Irish emergency room nurses, those who perceive their relationships with physicians to be collegial (i.e., having a good relationship) feel significantly less emotionally exhausted and less depersonalized (91).

#### Relevant Supportive Relationships

##### Peers

In a large sample size study in Belgium emergency room nurses reported higher social support from colleagues than from their nurse supervisor. However, when they received support from the supervisor, nurses indicated less psychosomatic distress (92, 93). A large sample size study examined the social network integration in relation to resilience of Taiwanese emergency room nurses who had experienced verbal or physical violence by patients or their families. In this research resilience is regarded as a process of adapatation to adverisity or stressful events, and it helps individuals cope with challenges in life and recover from trauma. The results showed that among all measured forms of social network (i.e., family, peers, and religious groups) only peer support enhanced resilience among abused nurses (94).

In contrast, another large sample size study among emergency department personnel, compared 356 doctors with 279 nurses, and showed that low social support among coworkers was associated with poor mental health only among doctors, but not among nurses (95). However, the sample frames of the two Belgian studies are unclear since 50–58% of these emergency room nurses also functioned as paramedics in ambulance care.

##### Supervisors

In the Belgian study mentioned above, lack of social support from the nurse supervisor for emergency room nurses was referenced as an important predictor of psychosomatic distress (92, 93). In Spain, low social support from nurse supervisors for emergency room nurses was associated with high scores of emotional exhaustion (95).

In a study among 11 emergency departments in Belgium, 292 emergency room nurses were studied on the association between social support from supervisors and nurses' turnover intention. This association was only significant among female nurses whose turnover was higher due to emotional exhaustion (96).

### Operating Room Nurses: Perioperative Nurses and Nurse Anesthetists

Work in operating rooms differs from other clinical areas. While nurses in other settings focus their attention on direct patient care and planning, operating room nurses provide direct patient care only briefly prior to anesthesia. Frequently, sometimes up to 8 or 12 continuous hours, time is spent one-to-one with a physician while performing a surgery. Compared to nurses on medical wards, operating room nurses do not experience stress as much from involvement with patients and their families, but mostly from interacting with physicians (97). In fact, here interpersonal relationships are the most frequent source of stress (97, 98).

In operating rooms two nurse specialties are distinguished: a perioperative nurse in surgical teams, and a nurse supporting the anesthesia team. In a study among Finnish operating room nurses, workplace culture was investigated through the constructs of job stress, job satisfaction, and practice environment in the operating room. The demands and content of the work of anesthesia or perioperative specialties seem to determine differences in workplace cultures and in particular the experience of job stress (99).

Study results for perioperative nurses and anesthesia nurses will be summarized separately below.

### Perioperative Nurses

#### Study Characteristics

Of all the identified articles on the mental health of nurses working in surgical teams (21), eight addressed social connections in the environment (38%). Five studies were quantitative, two qualitative, and one was mixed. Six studies focused on general embeddedness, i.e., social networks, relationship with the surgeon, workplace violence, and bullying. Two studies focused on relevant supportive relationships within the organization, i.e., coworkers, supervisors, or nursing manager.

#### General Embeddedness

Social connections may not only diminish the impact of job-related stress but could also be a source of stress. The operating room is a unit in the hospital where bullying seems to be common practice (100, 101), though it does not automatically lead to low job satisfaction (102, 103). A study among perioperative personnel in two different hospitals in the U.S. indicated that personnel did not perceive this disruptive behavior as problematic, yet the results do show a positive correlation with emotional exhaustion (102). In this study, however, there may be a potential source of non-response bias (29% response rate) and unclear sampling (53% surgical technologists, 45% nurse). In a large sample size study among different nursing units in Korea, physicians in the operating rooms were reported as the most frequent perpetrators of verbal abuse and sexual harassment (104). A qualitative study in Canada focused on the contribution of specific factors, such as an aura of seclusion, an inability to leave and hierarchy, to physician-perpetrated abuse in the operating room. All of these factors fostered a setting conducive to abuse without consequences. The (10) interviewed operating room nurses experienced negative psychological and social health effects from the abuse (105).

A qualitative study from the U.K. found that (predominantly female) nurses perceive “looking after surgeons” to be one of their stressful responsibilities. This “hostess” role is a kind of emotional labor but performed with coworkers rather than patients. Seventeen interviewed operating room nurses felt this emotional labor was necessary to minimize emotional disturbances of the surgeons (106).

#### Relevant Supportive Relationships

##### Peers

Members of surgical teams depend on each other for the performance of their functions. In a large sample size Australian study, Gillespie (107) measured the degree of collaboration and support among nurses in the operating room in relation to their resilience, and found it to be limited and not to be a statistically significant explanatory variable of self-reported “resilient qualities” of operating room nurses. However, the total sample of 772 respondents in this study included 415 perioperative nurses, 69 nurse anesthetists and the rest were other professionals working in the operating room. We should refrain from conclusions about perioperative nurses in this study due to this unclear sampling frame.

In another Australian study, Michael (108) examined social support within the operating room following a traumatic event. The amount of social support from perioperative coworkers was higher than the amount from supervisors. Coworkers also dealt more effectively with the problem and the respondents' feelings.

##### Supervisors

In the same study perioperative nurses reported a low level of support from supervisors (i.e., nursing manager) following a traumatic event (108).

### Nurse Anesthetists

#### Study Characteristics

Of all the identified articles on the mental health of nurses working in anesthesia teams (24), four addressed social connections in the environment (17%). Two studies were quantitative, one qualitative and one is mixed. All four studies focused on general embeddedness, i.e., social networks, interpersonal relations or conflicts, workplace aggression, and incivility. No research is found on relevant supportive relationships in the environment in which the nurse anesthetist is embedded.

#### General Embeddedness

The direct supervisor of the nurse anesthetists in the operating room is not the surgeon but the anesthesiologist. Nurse anesthetists and anesthesiologists have many overlapping skills. Therefore, the allocation of work tasks can be a source of conflict and interpersonal stress (98, 109, 110).

Among U.S. nurse anesthetists, interpersonal work relationships caused more stress than any of the other perceived job stressors, such as workload or production pressure (111). Nurse anesthetists' workplace stress was significantly correlated with experiences of aggression (80%) by physician supervisors (58.4%) and coworkers (36.6%) (112). For Dutch nurse anesthetists, conflicts with coworkers were strongly related to job stress and the intention to leave (109).

Elmblad et al. (113) focused their research on the effects of incivility, i.e., rude or disruptive behaviors, as well as on distress and burnout. They define burnout as a state of prolonged physical and psychological exhaustion, which is perceived as related to the person's work. The researchers found a direct and statistically significant relationship between workplace incivility and burnout. The sources of incivility affecting nurse anesthetists in the U.S. are general hospital employees, patients, visitors, and physicians; a lesser prevalent source of incivility were other nurse anesthetists. The least prevalent source of incivility were direct supervisors (i.e., the person she reports to most frequently) of nurse anesthetists. It is unclear from the used questionnaire in this study if the physician mentioned was the surgeon or the anesthetist (or both), and if the direct supervisor was the nurse manager or anesthetist. A direct and statistically significant, relationship existed between workplace incivility and burnout.

### Intensive Care Nurses

#### Study Characteristics

The search identified 199 articles on the mental health of intensive care nurses, of which eight addressed social connections in the environment (4%). Six studies were quantitative and two were qualitative. Six out of these eight studies focused on general embeddedness, i.e., social work environment, communication (114) cultural context and the relationship with physicians. Three studies focused on relevant supportive relationships within the organization, i.e., supervisors (115, 116) and peers (115). One study also expanded its focus on supportive relationships at home (115).

#### General Embeddedness

A small sample size study in the U.S. compared nurses in two general medical units with those in two ICUs. The study showed that psychological symptoms were not affected by the major patient-care activity of a given unit, but rather by social system variables, including peer cohesion and staff support (117). Mrayyan (118) conducted a larger sample size comparative study on job stressors in ICUs and wards. ICU nurses reported higher numbers of conflicts with physicians than the general wards nurses. Mahon (119) examined through a critical ethnography the cultural context of a Canadian Pediatric Intensive Care Unit (PICU). The lack of respect shown to nurses by hospital administrators appears to be one of the major sources of stress. Many actions or demands, such as “floating” nurses to other units while the PICU was quiet, appeared to undermine the sense of team and belonging, which is fundamental to PICU nurses' retention. An institutional ethnography, also conducted in a Canadian PICU, showed that negotiating patient care within the hierarchy (from a lower position than the physicians) was a consistent source of stress for the nurses (120). Brazilian nurses who have poor relationships with physicians, experienced higher levels of emotional exhaustion. This negatively influences their perception of quality of care, job satisfaction and intention to stay in their jobs (121).

#### Relevant Supportive Relationships

##### Peers

Mrayyan (118) found that ICU nurses score higher on social emotional support behaviors toward others nurses experiencing stressful situations than nurses working at wards.

### Police Officers

#### Study Characteristics

Of all the identified articles on the mental health of police officers (172), nine addressed social connections in the environment (5%). Eight studies were quantitative, of which one included a longitudinal design, and one study was qualitative. Eight of the nine studies focused on general embeddedness, i.e., relationships with coworkers (122) social capital at work and at home, social network, marital functioning (123) workplace incivility, bullying (124) and social undermining. Two studies (also) focused on relevant supportive relationships whitin the organization, i.e., peers and supervisors.

#### General Embeddedness

A study among U.S. police officers showed that good and effective cooperation between units as well as trust in work partners (i.e., “social capital within a work environment”) can lower the negative aspects of stress. Even when officers reported high levels of potentially traumatic events, strain levels did not increase if there was a certain degree of social capital within the police unit. The latter was the most significant factor in reducing the negative effect of stress, closely followed by “stability at home” (i.e., support from family or friends and talking about problems with spouse, relatives or friends) (125). Among Nigerian police officers, lower scores on “workplace social capital” were related to anxiety and depression symptoms (126).

Adams and Buck (127) compared the stressfulness of interactions with civilians and suspects (both outsiders), with workplace incivility from coworkers and supervisors (both insiders) among US police officers. It turned out that the average stress arising from insiders was similar to those from outsiders. Both stressors were positively related to psychological distress, emotional exhaustion, and turnover intention. A study among Slovenian police officers focused on behavior intended to hinder, over time, the ability to establish and maintain positive interpersonal relationships, work-related success, and favorable reputation. This social undermining by coworkers and/or supervisors was significantly, negatively, linked with employee mental health (128).

A large network (at work, home and/or community), in terms of frequency of contact and level of satisfaction, was not sufficient to prevent the development of burnout (emotional exhaustion, depersonalization and lack of accomplishment) among Mexican traffic police officers (129).

#### Relevant Supportive Relationships

##### Supervisors

The researchers of the previously mentioned study among Slovenian police officers found that the negative effects from social undermining by coworkers could only modestly be compensated by social support from supervisors. When supervisors were the source of undermining, as well as the source of social support, police officers report significantly more psychosomatic symptoms (128).

Buunk and Verhoeven (130) conducted a fine-grained analysis of stress-reducing features of daily social interactions, over 1 week, among Dutch police personnel. They found the degree of intimate support provided by supervisors closely related to less stress at the end of the workday.

##### Peers

Buunk and Verhoeven also found that a higher frequency in social interactions with peers (labeled as “intimate support”) was linked with more reported negative affect at the end of the day (130).

### Military Personnel

#### Study Characteristics

Of all the articles found on the mental health of military personnel (825), 35 addressed social connections in the environment (4%). All studies were quantitative, except for two qualitative studies. The majority (33) of these studies focused on general embeddedness under the following construct; unit cohesion (131) or group (132) cohesion (133) camaraderie (134) family cohesion or family conflict, a sense of community or belongingness (135–137) social connectedness (138) community integration or social network (139). Three studies focused on relevant supportive relationships, two inside the organization (i.e., from the supervisor) and one included non-military relationships (friends and family).

#### General Embeddedness

A sense of belonging may protect service members from depression and posttraumatic stress, starting from pre-deployment preparations through deployment and post-deployment adjustment (140). The term deployment is used by the military to describe sending troops to carry out a combat, peacekeeping, or humanitarian mission. Among Air Force men above the age of 29 years, who have been exposed to high levels of combat, a strong sense of belonging protected against suicidal ideation (141).

In military studies, unit or group cohesion was a topic of considerable interest and often used interchangeably with social support. However, unit cohesion is a multidimensional construct. It may include not only interpersonal aspects, such as emotional bonds among unit members (i.e., social cohesion), but also task-oriented aspects, such as shared commitment to a goal or mission (i.e., task cohesion) (142). Research results among personnel of the Canadian Army and Navy Forces highlighted the importance of this distinction. Where task cohesion seems to be critical to the operational effectiveness, social cohesion was related to reduced psychological symptomatology (143).

This finding was confirmed in a study among Australian military personnel deployed in the Middle East. Respondents with low unit (social) cohesion had higher odds of PTSD symptoms compared with those reporting high unit cohesion (144). Among U.S. Army soldiers, the confirmed link between exposure to stressors before deployment and PTSD symptoms after deployment (33) appears to be weaker when unit support was high (145). In contrast, another study found unit cohesion not to be related to post-deployment mental health outcomes. However, when an individual perceived their unit cohesion as lower compared to other members of the same unit, there was a significant association with post-deployment PTSD and depression symptoms (146).

Contrary to these findings, a study among Vietnam combat veterans found that low to moderate unit cohesion is related to lower reported rates of PTSD symptoms. Very high levels of unit cohesion was associated with higher than expected levels of PTSD symptoms (147).

Among regular U.K. military personnel, comradeship (i.e., “I felt a sense of comradeship between myself and other people in my unit”) was associated with greater alcohol abuse (148). Comparable results were found among U.S. Marines where higher unit cohesion was associated with a higher likelihood of alcohol misuse (146). Problematic alcohol use is well-known to be linked to posttraumatic stress reactions (149).

Among female service members and veterans, sexual harassment (by unit leaders or other unit members) during deployment was more highly associated with post-deployment PTSD symptoms (150) than combat exposure (151). One study concluded that it is not the culture or unit cohesion but the tolerance of sexism in the organization that accounts for the levels of harassment (152).

The following studies focused on the mental health of military personnel post-deployment (i.e., veterans). In most U.S. studies the status of “veteran” includes personnel who returned from deployment, who still serve and may be deployed again. However, in Europe the status of “veteran” is defined as former defense personnel (153).

U.S. army soldiers with higher than average unit cohesion during deployment and with post-deployment community support, had significantly lower post-deployment PTSD scores (154) and fewer suicidal thoughts (155). Formerly deployed U.S. veterans with low postemployment support scored high on PTSD and suicidality (156). A study among U.S. veterans returning from Middle East underscores this effect of post-deployment social support and reintegration on mental health symptoms (150).

In a qualitative study in Britain, 25 World War II veterans were interviewed about their social support experiences both during the war as well as during the years afterwards. There was a clear distinction: Wives and families generally provided practical and emotional help that is not associated directly with the traumatic memories, whereas comradeship (with fellow soldiers of the same generation) and veterans' associations provided the means to process traumatic experiences (157).

Among U.S. female active duty members who experienced social conflict during post-deployment, some of the benefits that social support might provide, were canceled out. Instead, more symptoms of PTSD and anxiety were reported (158). A lack of family cohesion, the degree to which family members are helpful and supportive, emerged as a consistent predictor of PTSD diagnosis for U.S. troops who served in the Persian Gulf War (159). Israeli soldiers who returned after war, and who were diagnosed with delayed PTSD, are less socially integrated in their community. That is, they regard their ties to their families as weak or non-existent (160). Later studies confirmed this notion and found that their family environment was less cohesive than those of the control groups (161, 162).

When service leavers of the U.K. military maintain a social network in which most members are still in the military, or if they belong to fewer social organizations outside the military and participate in fewer social activities outside work, higher levels of mental problems were found (163). Among United Nation soldiers who served in former Yugoslavia, the only statistically significant moderator between trauma exposure and trauma symptoms is the available support from a social network of friends and colleagues. Family and neighbors, on the contrary, seemed to be relatively unimportant as buffers (164). Unfortunately, the instrument in this study does not distinguish between friends at home vs. friends among colleagues. A large sample size study among U.S. Air Force personnel showed that PTSD symptoms were less likely when service members experienced support from family, neighbors as well as their community (165).

In contrast to these research findings, a study among U.S. millennial veterans who were transitioned back to their communities, found that a high sense of community is not a protective factor for mental health or reintegration. In fact, to the surprise of the researchers, results suggest that veterans who are connected to their local communities may be more at risk for reintegration difficulties and depression (166).

A qualitative study among Dutch combat veterans indicated that societal misrecognition may directly or indirectly contribute to moral injury (167).

#### Relevant Supportive Relationships

##### Supervisors

The association between stress exposure in prior life and PTSD symptoms after deployment (33) appeared to be weaker when U.S. Army soldiers' satisfaction with leadership is high (145). Among U.K. troops who were deployed in Iraq, perceived interest from seniors was associated with a lower probability for PTSD and other mental disorders (148).

##### Home

Deployed U.S. military personnel reported similar levels of support from military and non-military sources. However, only the social support from non-military friends and family had a positive influence on overall mental health (168).

### Synthesized Findings

#### Quantitative

Of all screened studies which focused on mental health of high-risk professionals, only 2–7 percent address social connections and relationships in the environment, with the exception of the nurses in operating rooms. In the studies of operating room nurses, a higher percentage focused on social connections and relationships (nurse anesthetists 17%; perioperative nurses 38%), though the total number of studies is considerable lower (45 studies) in comparison to other high-risk professionals (i.e., from 103 studies of emergency room nurses to 825 studies of military personnel).

The eligible studies can be distinguished as follows: The majority focused on the connections in the environment, i.e., general embeddedness, whereas a minority (0–36%) focused on relevant supportive relationships among these connections.

Studies on general embeddedness focused primarily on the organization. Only a minority of studies focused on social connections outside the organization (i.e., only one study of police officers, paramedics and intensive care nurses and two studies of firefighters). In contrast, 23 out of 35 studies among soldiers and other military personnel focused on social connections (general embeddedness) outside the military organization, i.e., friends, family, and their civilian community.

#### Qualitative

The included studies of **firefighters, paramedics**, and **emergency room nurses** showed that being embedded in their social environment can be a protective factor against mental health problems. Peers can be an important source of support for these professionals, but relationships with supervisors seem to be more important, especially for firefighters and emergency room nurses. Studies of **perioperative nurses** and **intensive care nurses** are inconclusive as to which supportive relationships in their environment are relevant to help them deal with potentially traumatic events. However, study results show that their connections with others in their work environment can also have a negative influence on their mental health. **Nurse anesthetists'** connections at work can be source of stress. We found no research addressing supportive relationships in their environment. Study results for **police officers** are inconclusive and show that social connections in their work environment can have positive as well as negative effects on their mental health. Supervisors seem to offer relevant supportive relationships.

Lastly, **military personnel** studies show that being embedded in their work environment may be a protective factor against mental health disorders. Despite the general positive overtones, in some studies these connections with others are also associated with heightened PTSD levels and alcohol misuse. A supportive relationship with their supervisor can protect against PTSD. While, the study results are inconclusive about the protective effect of being embedded at home and in their community, one study also showed that supportive relationships with friends or family members have a positive effect on their overall mental health.

On a **general note**, several methodological challenges, such as small or unclear samples, limit the interpretation of some studies. Considerable diversity exists in the conceptualization and measurement of, for example, social support by different researchers. Unfortunately, many researchers used scales that were created *post-hoc* from large data sets or created their own scales without psychometric testing or development. Other researchers used single-item measures. Although most of these scales have some face validity, they almost certainly have low reliability.

### Summary of Main Findings

Of the 9,047 screened studies 1,705 focused on the professionals' mental health, a minority (89) of which addressed social connections or relationships in the environment. In studies to date on high-risk professionals, the role of the social environment in relation to traumatization and lower level of mental health is still relatively neglected. A qualitative analysis of the eligible studies shows that in most occupational groups being embedded in a social environment may protect professionals against mental health problems. However, in some occupations, study results are inconclusive, and in some occupations social connections may be additional stressors or even damaging to mental health. Additionally, the source of relevant supportive relationships (peer, supervisor or friend, family member) shows great variety across and even inconsistencies within occupational groups.

## Discussion

The past few decades witnessed a growing concern about professionals who are called to help and save others on a daily basis, while risking their own (mental) life. This may result in trauma-related mental health problems and ultimately in the intention to leave their profession. Trauma researchers and theorists have extensively studied independent, intervening, and moderating variables that may affect the risk of traumatization. Consequently, an enormous amount of studies resulted in considerable insight in personal factors. However, traumatization is also related to peoples' social environments. More importantly, what happens after a potentially traumatic event may have stronger impact on mental health than the experience itself, as is well-known and documented by trauma therapists (33, 169–171). Traumatized individuals often speak of the devastation caused by “secondary wounding” when they perceive that others (in their social environment) blame them, withhold validation, denigrate the severity of their experience, or otherwise contribute to the shaming of their symptoms (172, 173). Nevertheless, the majority of research still tends to limit analysis of the risk of traumatization to the individual, while largely neglecting the social context of the person, i.e., the social environment in which he is embedded.

Being socially embedded provides a lens through which people interpret daily incidents (45), and may therefore influence *how* potential traumatic incidents are interpreted and processed, or not. Different occupational groups may have different sources of supportive relationships, which may all in different contexts and stages be more or less effective in ameliorating stress. These sources may indeed shift over time. For example, among military personnel it can shift from work-based support (e.g., from fellow military personnel and supervisors) to post-deployment support (e.g., from family and friends) once individuals return home.

Generally, the sense of connection and belonging to an organization, home or the community, satisfies basic needs for affiliation, security and identity (174). However, social relationships are only supportive in as far as they are non-conflictual, imply care for the well-being of those involved and facilitate trust. It is of great importance for high-risk professionals, to work with the conviction that others “have your back”; to be confident of the availability and help of others, to feel assured of emotional support, especially in their most vulnerable moments.

This support is not a given in every professional group. As this review demonstrated, interpersonal relationships can be a double-edge sword, with aspects of support as well as conflict. Work relationships that generate stress are most prevalent among specialized (OR and ICU) nurses and amongst police officers as well. We might suppose that in specialized and “enclosed” hospital wards, such as ORs and ICUs, coworkers are physically in close contact to offer emotional support when they experience stressful situations. However, interpersonal conflict, disruptive behaviors and bullying seem to be common practice among these professional groups, and also create hostile work settings (100, 101). This may continue for a long time, without consequences, due to the hierarchical nature of the work setting and the (physical) inability to leave the setting. Among police officers social undermining, another negative workplace behavior, was prevalent. Among military personnel sexual harassment was found.

Negative interpersonal experiences are a considerable source of stress (61). They disrupt social networks (175) and cancel out the positive effect of social support (170). This finding can be especially detrimental for high-risk professionals, given that they often have to rely on each other and that they are an important source of social support for one another (14, 69, 80, 83, 92, 108, 145, 148, 176). A meta-analysis shows that social support deficiencies in military samples are associated with more psychological effects than among civilian samples (33).

In addition, the experience of inconsistent responses, i.e., support from as well as conflict with the same person, leads to feelings of insecurity, and lack of control, trust, and predictability in the relationship. This is also associated with higher levels of personal distress (128, 177) and might increase the risk of traumatization (178). While studying persons who frequently face potential traumatic situations, or when treating high-risks professionals with trauma-related disorders, we should not overlook these “dark sides” of social relationships (61) (p. 147).

Embeddedness, whether with supportive or damaging relationships, may be particularly salient during the organizational or professional socialization process, which in many high-risk occupations takes place during the formative late adolescent years. In emergency organizations knowledge and practices are mainly experience-based and “on-the-job training” in the existing culture is prominent. More importantly, mentors (senior colleagues and supervisors) not only teach novices professional norms, but also teach coping (or survival) strategies (179, 180) when dealing with potential traumatic events. Coping often occurs in response to stressors that arise in situations where other people are also affected and attempt to cope with them. This means that the coping actions of one person may be constrained, encouraged, or channeled by the expectations and actions of others (181). Young professionals may be particularly prone to incorporating others' coping strategy, adaptive or not, when they enter a novel environment, as they are highly motivated to belong in order to develop a social (and professional) identity. This may make them vulnerable in the long run, as adaptation is always a product of complex interactions between an individual and his social environment. A certain coping response of an individual may be considered adaptive or helpful in one environment or situation, but may be maladaptive or inappropriate in another (181). A certain social environment may promote an individual's willingness to talk about a traumatic event or, on the contrary, it may inhibit the expression of emotional problems (182–185). Likewise, people may receive social support when the social environment acknowledges a crisis, but support may be withheld in situations which are considered to be shameful (186–188). In short, even though supportive relationships are vehicles for psychological development and essential for sustaining mental health (34), they are highly dependent upon and tailored to the social environment in which a young professional is formed.

## Limitations of this Review

Several limitations of our systematic literature review may provide additional research opportunities. First, stressors among high-risk professionals may involve a wide variety of factors such as conflicts between work and home, role conflicts or spill-over effects. These conflicts are typically called “inter domain” conflicts, and consequently in our study we struggled in the selection process with the question to which domain stressors should be attributed. Any comprehensive inquiry into the social context must map the professionals' larger social field to ensure that all potential relevant environmental factors are taken into account.

Second, the “sense of community” construct is often compared to constructs found in industrial-organizational psychology, e.g., organizational commitment and job satisfaction (74). Although we decided to exclude studies that solely focused on the construct “job satisfaction,” we are aware that it might be (indirectly) related to retention. Lastly, we excluded studies that solely focused on perceived social support. We maintain that perceived social support is primarily to be seen as a personal factor. However, indirectly environmental factors play a role as well as perceived social support is influenced by personal generalized assumptions that emerge from numerous previous experiences with help offered by others (61, 189, 190).

## Further Research

The initial aim of this review was to explore the influence of social connections and relationships in the professionals' environment on traumatization. However, excluding studies focusing on other mental health issues while only including studies on traumatization, yielded considerably fewer eligible studies (i.e., 27 military-studies, 5 firefighters studies, 1 each for police, ICU, perioperative nurses, ER-nurses, and zero for nurse anesthetist and paramedics). An explanation may be that the construct “trauma,” or even the use of the very word “trauma,” may be stigmatized (191–195), which may direct research away from PTSD toward other mental health problems such as substance abuse, burnout, depression, or moral injury (4, 196). Some of those mental disorders have comorbidity with PTSD (7, 8). All mental health problems, however, have in common that the mental capacity to integrate stressfull events is compromised (12, 13). Being aware of the broad range of trauma-related mental health problems (7, 8) can assure that we, researchers and clinicians, do not lose sight of the fact that these professionals are frequently exposed to potentially traumatic events.

The findings and implications from studies among one specific occupational group have often been applied to other professions, such as the findings among firefighters to paramedics or other “emergency services personnel.” Take for example Australian “ambulance officers”: it is not clear whether they resemble paramedics, or whether they more closely resemble EMTs and ambulance drivers who each have different skills and training backgrounds. Variables such as the type of training received, the nature of the traumatic exposures, and the probability of repeated exposures, as well as contextual variables, especially their primary sources of support, might differ from one profession to the other. It may require careful ethnographic research to judge to what extent different high-risk professions may be comparable in terms of traumatization.

Most studies in this review have used quantitative self-report measures to investigate embeddedness, enacted social support and their related constructs. Although a perception most likely reflects reality, self-report measures are subject to a variety of biases (44, 197). The diversity of social relationships suggests that much could be gained from using diverse research methods, including qualitative approaches (198). This type of research can provide critical directions for future quantitative studies, for it may result in more meaningful hypothesis about the influence of social relationships on the risk of traumatization.

## Author Contributions

RG and MV contributed conceptualization, formal analysis, and methodology and of the review. RG wrote the first draft of the manuscript. All authors contributed to manuscript revision, read, and approved the submitted version.

## Conflict of Interest

The authors declare that the research was conducted in the absence of any commercial or financial relationships that could be construed as a potential conflict of interest.
