# Supplementary material for: Social Embeddedness of Firefighters, Paramedics, Specialized Nurses, Police Officers, and Military Personnel: Systematic Review in Relation to the Risk of Traumatization
Source: Front Psychiatry. 2020 Dec 21;11:496663. doi: 10.3389/fpsyt.2020.496663 (PMC7779596; doi:10.3389/fpsyt.2020.496663)
Supplement: Supplementary file 2 [file Data_Sheet_2.pdf]

Data sheet 2 Major Search Terms

(String: major term 1 OR synonym) AND (major term 2 OR synonym) AND (major term 3 OR synonym)

P. Professional domain

P1 Major term: Firefighters

Related terms: fire service workers, firefighter, fire fighter, fire-fighter, urban fire service, volunteer firefighter, volunteer fire fighter, volunteer fire-fighter.

P2 Major term: Ambulance Personnel

Related terms: on-road ambulance officers, ambulance officers, ambulance personnel, ambulance worker, ambulance-men, ambulance service, paramedics, emergency medical technicians, EMT, emergency responders, or paramedic.

(Related terms: First responders, first-responders)

P3 Major term: Emergency Room Nurses

Related terms: ED nurses, ED personnel, emergency room nurses, ER nurses, ER personnel, Emergency nurses, emergency personnel, emergency services personnel, emergency service personnel, ems personnel, emergency workers.

P4 Major term: Perioperative nurses

Related terms: operating theatre nurses, scrub nurses, operating theatre personnel, operating room personnel, OR, operating room nursing.

P5 Major term: Nurse Anesthetists

Related terms: nurse anesthesia, nurse anaesthesia, anesthetic assistants, anaesthetic assistants (not recovery, stricted to OR)

P6 Major term: Intensive Care nurses

Related terms: critical care nurses, high dependency nursing, ICU, intensive care personnel.

P7 Major term: Police officer

Related terms: police officers, law enforcement, law enforcement.

P8 Major term: Military personnel

Related terms: soldiers, combat, veteran, Armed Forces, combat veterans.

*(*active military service)

O. Mental well-being

Major term: Trauma

Related terms: stress, stress disorders, psychological stress, acute stress disorders, posttraumatic stress disorder, posttraumatic, post-traumatic, posttraumatic reactions, PTSD, trauma, acute posttraumatic stress disorder, chronic posttraumatic stress disorder, stress-related, distress, care-giver stress, occupation stress, work-related stress.

Major term: Mental health problems

Related terms: psychological well-being, psychological strain, depression, burnout, burn out, burn-out, anxiety, hostility, aggression, emotional symptoms, healthy adjustment, disturbed sleep, somatization, somatisation, alterations in work function, difficulties with interpersonal relationships, increase in substance use, worry, health risk behaviors, psychiatric symptoms, emotional symptoms, cognitive, psychiatric diagnosis, psychopathology, self-efficacy.

Major term: Retention

Related terms: sick leave, performance, absenteeism, work absence, satisfaction, job satisfaction, turnover intention, turnover rate, intention to remain, work leave.

(Related: workplace adversity, occupational stressor)

I. Social Environment

Major term: Social support

Related terms: perceived social support, enacted social support, psychosocial support, organizational support, organisational support, social-support networks.

Major term: Social embeddedness

Related terms: social connectedness, social network, unit cohesion, work cohesion, cohesion, social interaction, teamwork, Interpersonal, intergroup relations, inter-organizational, inter-organisational, organizational communication, organisational communication, communication, organizational socialization, organisational socialisation, organizational, organisational.

Major term: Sense of community

Related terms: psychological sense of community, belongingness, attachment, kinship, bonding, relation, relations, relational, affective commitment, commitment, organizational commitment, organisational commitment, engagement, engagement at work, work engagement, employee engagement, employee loyalty, job engagement, nurse engagement, personal engagement, families, spouses, wives, domestic strain, marital interaction, peer group, collectively, connectedness.
