# Supplementary material for: Social Embeddedness of Firefighters, Paramedics, Specialized Nurses, Police Officers, and Military Personnel: Systematic Review in Relation to the Risk of Traumatization
Source: Front Psychiatry. 2020 Dec 21;11:496663. doi: 10.3389/fpsyt.2020.496663 (PMC7779596; doi:10.3389/fpsyt.2020.496663)
Supplement: Supplementary file 3 [file Data_Sheet_3.pdf]

Data Sheet 3 Search Strings

Search Strings MEDLINE

Firefighters
"fire service workers"[All Fields] OR "firefighter"[All Fields]) OR "fire fighter"[All Fields]) OR "urban fire fighters"[All Fields]) OR "urban firefighters"[All Fields]) OR "volunteer firefighters"[All Fields] OR "volunteer fire fighters"[All Fields]) OR "fire man"[All Fields] OR "volunteer fireman"[All Fields] OR "fire men"[All Fields] OR "volunteer firemen"[All Fields] OR "firemen"[All Fields]
AND
"psychological"[All Fields] OR "mental"[All Fields] OR "psychiatric"[All Fields] OR "sick/absent"[All Fields] OR "turnover/absenteeism"[All Fields] OR "satisfaction"[All Fields] OR "workplace adversity"[All Fields] OR "resilience"[All Fields] "stress"[All Fields]

Ambulance personnel
"ambulance officers"[All Fields] OR "ambulance officer"[All Fields] OR "ambulance personnel"[All Fields] OR "ambulance workers"[All Fields] OR "ambulance worker"[All Fields] OR "ambulance men"[All Fields] OR "ambulance service"[All Fields] OR "paramedic"[All Fields] OR "paramedic/ambulance"[All Fields] OR "emergency medical technician"[All Fields] OR "emergency medical technician paramedics"[All Fields] OR "emergency medical technician paramedics emt ps"[All Fields] OR "paramedics"[All Fields]
AND
"psychological"[All Fields] OR "mental"[All Fields] OR "psychiatric"[All Fields] OR "sick/absent"[All Fields] OR "turnover/absenteeism"[All Fields] OR "satisfaction"[All Fields] OR "workplace adversity"[All Fields] OR "resilience"[All Fields] "stress"[All Fields]

Emergency Room Nurses
"emergency room nurse"[All Fields] OR "emergency room nurses"[All Fields] OR "emergency room personnel"[All Fields] OR "emergency nurse"[All Fields] OR "emergency nurses"[All Fields] OR "emergency nurse"[All Fields] OR "emergency department nurses"[All Fields] OR "emergency department personnel"[All Fields] OR "emergency personnel"[All Fields] OR "emergency service personnel"[All Fields] OR "emergency workers"[All Fields]
AND
"psychological"[All Fields] OR "mental"[All Fields] OR "psychiatric"[All Fields]) OR "sick/absent"[All Fields] OR "turnover/absenteeism"[All Fields] OR "satisfaction"[All Fields] OR "workplace adversity"[All Fields] OR "resilience"[All Fields] "stress"[All Fields]

Perioperative Nurses
"perioperative nurse"[All Fields] OR "perioperative nurses"[All Fields] OR "operating theater personnel"[All Fields] OR "operating theatre nurse"[All Fields] OR "operating theatre nurses"[All Fields] OR "operating theatre personnel"[All Fields] OR "scrub nurse"[All Fields] OR "scrub nurses"[All Fields] OR "operating room nurse"[All Fields] OR "operating room nurses"[All Fields] OR "operating room personnel"[All Fields]
AND
"psychological"[All Fields] OR "mental"[All Fields] OR "psychiatric"[All Fields] OR "sick/absent"[All Fields] OR "turnover/absenteeism"[All Fields] OR "satisfaction"[All Fields] OR "workplace adversity"[All Fields] OR "resilience"[All Fields] "stress"[All Fields]

Nurse Anesthetists
"nurse anesthesia"[All Fields] OR "nurse anaesthesia"[All Fields] OR "nurse anaesthetists"[All Fields]) OR "nurse anaesthetist"[All Fields] OR "anesthetic assistants"[All Fields] OR "anaesthetic assistants"[All Fields] OR "anesthetic nurses"[All Fields] OR "anesthetic nurse"[All Fields] OR "anaesthetic nurse"[All Fields] OR "anaesthetic nurses"[All Fields] OR "anaesthetic assistant"[All Fields]
AND
"psychological"[All Fields] OR "mental"[All Fields] OR "psychiatric"[All Fields] OR "sick/absent"[All Fields] OR "turnover/absenteeism"[All Fields] OR "satisfaction"[All Fields] OR "workplace adversity"[All Fields] OR "resilience"[All Fields] "stress"[All Fields]

Intensive Care Unit Nurses

"intensive care nurses"[All Fields] OR "intensive care personnel"[All Fields] OR "critical care nurse"[All Fields] OR "critical care nurses"[All Fields] OR "intensive care nurse"[All Fields] OR "high dependency nursing"[All Fields]
AND
"psychological"[All Fields] OR "mental"[All Fields] OR "psychiatric"[All Fields] OR "sick/absent"[All Fields] OR "turnover/absenteeism"[All Fields] OR "satisfaction"[All Fields] OR "workplace adversity"[All Fields] OR "resilience"[All Fields] "stress"[All Fields]

Police officers
"police officer"[All Fields] OR "police personnel"[All Fields]) OR "law enforcement personnel"[All Fields] OR "law enforcement officer"[All Fields]
AND
"psychological"[All Fields] OR "mental"[All Fields]) OR "psychiatric"[All Fields]) OR "sick/absent"[All Fields]) OR "turnover/absenteeism"[All Fields]) OR "satisfaction"[All Fields] OR "workplace adversity"[All Fields]) OR "resilience"[All Fields] "stress"[All Fields]

Military personnel
"military"[Title/Abstract] OR "soldier"[Title/Abstract]) OR "combat"[Title/Abstract]) OR "armed force"[Title/Abstract] OR "combat veteran"[Title/Abstract] OR "military service members"[Title/Abstract]
AND
"psychological"[Title/Abstract] OR "mental"[Title/Abstract] OR "psychiatric"[Title/Abstract] OR "sick/absent"[Title/Abstract] OR "turnover/absenteeism"[Title/Abstract] OR "satisfaction"[Title/Abstract] OR "workplace adversity"[Title/Abstract] OR "resilience"[Title/Abstract] AND "stress"[Title/Abstract]
AND
"social support"[Title/Abstract] OR "social network/support"[Title/Abstract] OR "organizational support"[Title/Abstract] OR "unit cohesion"[Title/Abstract] OR "social interaction"[Title/Abstract] OR "socialisation"[Title/Abstract] OR "organizational"[Title/Abstract] OR "community"[Title/Abstract] OR "belongingness"[Title/Abstract] OR "relations"[Title/Abstract]) OR "camaraderie"[Title/Abstract] OR "military families"[Title/Abstract] OR "spouses"[Title/Abstract] OR "wives"[Title/Abstract] OR "connectedness"[Title/Abstract] AND "humans"[MeSH Terms]

Search Strings CINAHL

Police officers
police
AND
(MH "Psychology, Occupational+") OR (MH "Stress, Occupational+") OR (MH "Stress+") OR (MH "Stress Disorders, Post-Traumatic+") OR (MH "Personnel Retention") OR (MH "Personnel Turnover") OR (MH "Team Building") OR (MH "Teamwork") OR (MH "Workload")

Firefighters
firefighters
AND
(MH "Psychology, Occupational+") OR (MH "Stress, Occupational+") OR (MH "Stress+") OR (MH "Stress Disorders, Post-Traumatic+") OR (MH "Personnel Retention") OR (MH "Personnel Turnover") OR (MH "Team Building") OR (MH "Teamwork") OR (MH "Workload")

Ambulance personnel
(MH "Emergency Medical Technicians") OR ambulance personnel OR ambulance paramedics
AND
(MH "Psychology, Occupational+") OR (MH "Stress, Occupational+") OR (MH "Stress+") OR (MH "Stress Disorders, Post-Traumatic+") OR (MH "Personnel Retention") OR (MH "Personnel Turnover") OR (MH "Team Building") OR (MH "Teamwork") OR (MH "Workload")

Emergency Room Nurses
emergency room nurse OR emergency room nurses OR emergency department nurse OR emergency department nurses
AND

(MH "Psychology, Occupational+") OR (MH "Stress, Occupational+") OR (MH "Stress+") OR (MH "Stress Disorders, Post-Traumatic+") OR (MH "Personnel Retention") OR (MH "Personnel Turnover") OR (MH "Team Building") OR (MH "Teamwork") OR (MH "Workload")

Intensive Care Unit Nurses
'intensive care nurses'
AND
(MH "Psychology, Occupational+") OR (MH "Stress, Occupational+") OR (MH "Stress+") OR (MH "Stress Disorders, Post-Traumatic+") OR (MH "Personnel Retention") OR (MH "Personnel Turnover") OR (MH "Team Building") OR (MH "Teamwork") OR (MH "Workload")

Perioperative Nurses
perioperative nurses
AND
(MH "Psychology, Occupational+") OR (MH "Stress, Occupational+") OR (MH "Stress+") OR (MH "Stress Disorders, Post-Traumatic+") OR (MH "Personnel Retention") OR (MH "Personnel Turnover") OR (MH "Team Building") OR (MH "Teamwork") OR (MH "Workload")

Nurse Anesthetists
nurse anesthesist
AND
(MH "Psychology, Occupational+") OR (MH "Stress, Occupational+") OR (MH "Stress+") OR (MH "Stress Disorders, Post-Traumatic+") OR (MH "Personnel Retention") OR (MH "Personnel Turnover") OR (MH "Team Building") OR (MH "Teamwork") OR (MH "Workload")

Military personnel
(MH "Military Personnel+") OR (MH "Military Deployment+")
AND
(MH "Psychology, Occupational+") OR (MH "Stress, Occupational+") OR (MH "Stress+") OR (MH "Stress Disorders, Post-Traumatic+") OR (MH "Personnel Retention") OR (MH "Personnel Turnover") OR (MH "Team Building") OR (MH "Teamwork") OR (MH "Workload")

Last search: January 1-6th - 2019
