# Supplementary material for: Social Embeddedness of Firefighters, Paramedics, Specialized Nurses, Police Officers, and Military Personnel: Systematic Review in Relation to the Risk of Traumatization
Source: Front Psychiatry. 2020 Dec 21;11:496663. doi: 10.3389/fpsyt.2020.496663 (PMC7779596; doi:10.3389/fpsyt.2020.496663)
Supplement: Supplementary file 4 [file Table_1.docx]

Table 1 List of Eligible Studies: Studies referring to mental health of firefighters, paramedics, emergency nurses, perioperative nurses, nurse anesthetist, intensive care nurses, police officers, or military personnel in combination with social environment (social connections and/or supportive relationships).

| **RefNo.** | **Study Author/s**  **(Year)**  **Country** | **Journal** | **Method/Design** | **Sample** | **Construct** | **Measurements** | **Main findings** |
| --- | --- | --- | --- | --- | --- | --- | --- |
| ***Firefighters*** | | | | | | | |
| [70] | Wong et al.  (2014)  Taiwan | Revue Europeenne de psychologie appliquee | Quantitative: cross-sectional, *structural equation modeling* | 422 firemen | Job Stress / Work/Non-work conflict / Work/Leisure-conflict / Job demand/ Job control (autonomy) / **Job support (supervisors and co-workers)/ Family support** | Job Stress Scale (Netemeyer, House & Rizzo’s anxiety-stress scale) / WFC scales / WLC  Job Demand Scale / Work schedule autonomy scale / Manager support scale (4 items)  Developed: co-worker and family support scale (4 items) | The relationship between family support and job stress is fully mediated by work/family conflict.  Work/Leisure-conflict and Work/family conflict mediated partially the relationship demand, control, job support and stress.  RoB: developed scale not validated |
| [73] | Carey et al.  (2011)  USA | Journal of Occupational and Environmental Medicine | Quantitative: descriptive study | 112 professional firefighters in 6 different fire houses. | Sleep problems / Depression / Substance use (alcohol, caffeine, nicotine) **/ Social Bonding** / Quality of life (physical and mental well-being) | Pittsburgh Sleep Quality Index (PSQI) / Epworth Sleepiness Scale (ESS) / Beck Depression Inventory (BDI-II) / The Time Line Follow Back survey / Spirituality in Everyday Life (SEL): / Health Survey (SF-12) | Firefighters with poor mental well-being were more likely to report poor social bonding.  RoB: no psychometric testing of SEL |
| [74] | Cowman et al.  (2004)  USA | J Community Psychol | Quantitative: cross-sectional, *regression models* | 221 firefighters | **Psychological sense of community** / Social Support Networks/ Satisfaction and stress from providing care to others | Perceived Sense of Community Scale / Social Support Questionnaire (SSQ) / Caregiver Scale | Psychological sense of community mediated satisfaction support from coworkers and high levels of satisfaction are linked to less care-giver stress. |
| [75] | Stanley et al. (2018a)  USA | Psychological Services | Quantitative: cross-sectional | 840 firefighters | **Thwarted belongingness** / PTSD Symptoms | INQ: thwarted belongingness (TB-subscale) / PCL-Civilian | Greater belongingness and social support from family and friends is associated with less severe PTSD symptoms.  Social support from supervisors was significantly associated with lower overall PTSD symptom severity. |
| [76] | Stanley et al. (2018b)  USA | Psychiatry Research | Quantitative: cross-sectional, *regression models* | 1.131 firefighters (incl. 20 wildland firefighters) | **Thwarted belongingness** / Suicidal behaviors | SBQ-R / INQ | Thwarted belongingness (cf disconnectedness) was associated with lower levels of PTSD symptoms.  Feeling of disconnectedness was associated with higher suicide risk.  RoB: small subgroup sample |
| [77] | Tuckey & Hayward  (2011)  Australia | Applied Psychology: an international review | Quantitative: cross-sectional, path analysis | 547 volunteer Firefighters | Emotional demands /  Job resources (cognitive-emotional, emotional, physical/instrumental) /  **Camaraderie /**  Psychological Health Outcomes (traumatic stress symptoms, psychological distress, Burnout) | Demand-Induced Strain Questionnaire (DISQ) / 5 items scale occupational-specific resource, camaraderie (Sced, Bauer & Tuckey) / IES-R / General Health Questionnaire (GHQ) / Copenhagen Burnout Inventory | Camaraderie was a buffer against PTSD symptoms. |
| [78] | Armstrong et al.  (2014)  Australia | Aust J Psychol | Quantitative: cross-sectional, *regression models* | 218 firefighters | Post-trauma outcomes (PTSD, PTG) / Work context factors (operational and management hassles), **Sense of** **Belonging** / Social support / Coping | Impact of Events Scale-R (IES-R) / Posttraumatic Growth Inventory (PTGI)/ Operational and Organizational Police Stress Questionnaires (PSQ-Op + PSQ-Org) / Psychological Sense of Organizational Membership Scale (PSOM)/ 2-Way Social Support Scale / Coping Response in Rescue Workers (CRRW) inventory | Organizational belongingness did not predict reduction in PTSD symptoms. |
| [79] | Armstrong et al.  (2015)  Australia | Psychological Trauma: Theory, Research, Practice, & Policy | Quantitative: cross-sectional, *structural equation modeling* | 250 firefighters | Post-trauma outcomes (PTSD, PTG) / Operational and Organizational sources of stress / **Organizational belongingness** | IES-R / PTGI / PSQ-Op + PSQ-Org / PSOM | Organizational belongingness was a significant predictor of PTG. |
| [80] | Airili et al. (2014)  Finland | Work & Stress | Quantitative: two-wave 10-year longitudinal design | 403 professional firefighters | **Supervisory relations (supervisory support) / Interpersonal relations** (social support from colleagues), Task resources / Self-esteem / Work engagement / Work ability | Occupational Stress Questionnaire / Rosenberg Self-Esteem Scale / Utrecht Work Engagement Scale (UWES) / Work Ability Index (WAI) | Positive interactions between co-workers and support, and positive feedback from one’s supervisor had a positive effect on work ability (= health required for the job). |
| [81] | Lee et al. (2018)  Korea | American Journal of Industrial Medicine | Quantitative: cross-sectional, *regression models* | 6.369 firefighters | **Workplace discrimination** / Depression | Korean Working Conditions Survey (KWCS) / CES-D | 30.3% experienced workplace discrimination, and those had a higher likelihood of depressive symptoms. |
| [82] | Hom et al.  (2017)  USA | J Nerv Ment Dis | Quantitative: cross-sectional, *regression models* | 290 women firefighters | Sexual harassment / Capability of suicide / Anxiety sensitivity / Alcohol use / Depression / Perceived burdensomeness / **Thwarted belongingness/** Insomnia / PTSD/ Suicide risk | Acquired Capability for Suicide Scale-Fearlessness About Death (ACSS-FAD) / Anxiety Sensitivity Index-3 (ASI-3) / AUDIT-C / Center for Epidemiological Studies Short Depression Scale (CES-D) / Interpersonal Need Questionnaire (INQ) / Insomnia Severity Index (ISI) / PTSD Checklist (PCL)-5 / Self-Injurious Thoughts and Behavior Interview-Short Form (SITBI-SF) / Suicidal Behavior Questionnaire-Revised (SBQ-R) / Quality of Worklife Module (QWM) | Workplace (sexual) harassment is significant associated with higher levels of PTSD symptoms. |
| [83] | Bernabé & Botia  (2016)  Spain | J Health Psychol | Quantitative: cross-sectional, *descriptive analyses, structural equation analysis and hierarchical regression analysis* | 156 firefighters | Emotional demands /  **Emotional social suppor**t **behaviors** / Resilience /  Burnout (emotional exhaustion, cynicism)  Engagement (vigor, dedication) | Critical Incidents Scale / Emotional Social Support Scales for coworkers and supervisors (House & Wells) / Connor-Davidson Resilience Scale (CD-RISC) / Maslach Burnout Inventory (MBI-GS) / UWES | Emotional social support from supervisors and coworkers indirectly related to employee health, favoring resilience.  Bosses denoting support, recognition and social companionship were found to have an interaction effect on resilience. |
| [84] | Huynh et al. (2013)  Australia | J Occup Health Psychol | Quantitative: 1 year longitudinal design | 126 volunteer firefighters | Volunteer demands (emotional demands and work-home conflict) / Burnout (exhaustion and cynicism) / **Family and Friend support**/ **Organizational Connectedness** | Demand-Induced Strain Questionnaire /4-items Holohan & Gilbert’s …/ MBI-GS (adapted version) / Social Support Scale (4 items) / 15-item Four Dimensional Connectedness Scale | Support from family and friends protects against burnout, and moderates organizational connectedness. |
| ***Paramedics*** | | | | | | | |
| [69] | Lewig et al. (2007)  Australia | J Vocat Behav | Quantitative: Cross-sectional, *structural equation modeling* | 487 volunteer ambulance officer (VAO) | Job Demands (Time pressure, Work-Home Interference) / Job resources (Job control, **Peer support, Connectedness**) Burnout / Health problems / Determination to continue as a volunteer | Job Demands Scale (2 items) /  Holahan & Gilbert’s Work-home interference- Scale (4 adapted items) / Job Control Scale (1 item added on face validity) / Social Support Scale (4 items) / Volunteer Experience Survey: Connectedness scale (7 items) / MBI-GS: EE and Cynicism subscales / GHQ-12  1-item Determination to continue | Connectedness fully mediates the relationship between job resources and determination to continue. |
| [85] | Shakespeare-Finch & Daley (2017)  Australia | Psychological Trauma: Theory, Research, Practice, & Policy | Quantitative: Cross-sectional, *regression models* | 740 ambulance officers | Psychological distress  Resilience  **Workplace belongingness** | Kessler 10 (K10) / Brief Resilience Scale (BRS) / PSOM | Workplace belongingness was significantly associated with reduced distress levels and enhanced resilience levels. |
| [86] | King & DeLongis (2014)  Canada | Journal of Family Psychology | Quantitative: 4 workdays-longitudinal design | 87 couples *(in which 1 partner paramedic)* | Paramedics (perceived) works stress / Burnout / Coping (paramedic rumination, **spouses withdrawal)** / **Marital tension** | Perceived Stress Scale (PSS-4) / MBI-Human Services Survey (MBI-HSS):3 items / 3 items from Rumination-Reflection Questionnaire (RRQ) / Brief Ways of Coping - Interpersonal Withdrawal subscale (3 items) / Daily diary (marital tension-single item) | Significant associations between paramedics’ work stress and subsequent withdrawal. Paramedics’ work related burnout predicted withdrawal from their spouses. |
| [87] | Petrie et al. (2018)  Australia | PLOS One | Quantitative: Cross-sectional, *descriptive, regression analysis* | 1.622 ambulance personnel | Manager psychological safety climate/ **Management behavior** / Symptoms of common mental disorder/ Mental well-being | Psychosocial Safety Climate Scale (PSC-12); Management Support and Commitment & Management Priority subscales/ 9-items questionnaire manager behavior (developed)/ Kessler 6 (K6) / Short Warwick-Edinburgh Mental Well-being Scale (SWEMWBS) | Manager psychosocial safety climate had significant influence on levels of employee common mental health disorder symptoms, mental health disorder and well-being. |
| ***Emergency nurses*** | | | | | | | |
| [88] | Healy & Tyrell  (2011)  Ireland | Emerg Nurse | Quantitative: Cross-sectional, *descriptive survey* | Nurses (90) 87% and doctors (13) in 3 Emergency Departments | Workplace stress and experiences / **Employer (enacted) support** | *Questionnaire about stress (16 items)* | 74% reported not received social support from employer to help them deal with stress.  RoB: small sample size, no psychometric testing of questionnaire |
| [89] | Laposa et al.  (2003)  Canada | J Emerg Nurs | Quantitative: Cross-sectional, *correlational* | 51 Emergency Department personnel | PTSD symptoms / Sources of workplace stress / **Work-related responses to stress or trauma** | Health Professionals Stress Inventory (HPSI-R) / Posttraumatic Diagnostic Scale (PDS) / Work-related responses to stress or trauma: (6-items) | Interpersonal conflict was significantly associated with PTSD symptoms. 67% received inadequate support from administrators following a traumatic incident.  RoB: small sample size, no clear sample frame |
| [91] | O’Mahony  (2011)  Ireland | Emerg Nurse | Quantitative: Cross-sectional, *descriptive, simple linear regression analysis* | 64 Emergency nurses | Burnout / Work environment characteristics (**collaboration nurse-physician)** | MBI: EE, Depersonalization / NWI-PES | High levels of emotional exhaustion and depersonalization were significantly related to a having a good relationship with physicians.  RoB: small sample size |
| [92] | Adriaenssens et al. (2011)  Belgium | J Adv Nurs | Quantitative: Cross-sectional, *hierarchical regression analysis* | 254 Emergency nurses in 15 emergency departments | Job characteristics (job demand, control and **social support)** / Organizational variables (rewards, personal resources, material resources and procedures) / Job satisfaction / Turnover intention / Work engagement / Fatigue / Psychosomatic distress | Leiden Quality of Work Questionnaire for Nurses (LQWQ-N) / Checklist Individual Strength (CIS-20R) / UWES / Brief Symptom Inventory (BSI) | Social support by supervisors was a strong determinant of outcome (among lower turnover intention and psychosomatic distress).  RoB: unclear sample frame |
| [93] | Adriaenssens et al. (2015)  Belgium | J Nurs manag | Quantitative: Two-wave 18-months Longitudinal, *regression analysis* | 170 Emergency room in 15 emergency departments | Job characteristics (job demand, control and **social support)** / Organizational variables (rewards, personal resources, material resources and procedures) / Job satisfaction / Turnover intention / Work engagement / Fatigue / Psychosomatic distress | LQWQ-N / Checklist Individual Strength (CIS-20R) / UWES / BSI | Social support by supervisors was a strong determinant of outcome (lower turnover intention and psychosomatic distress) and this increased over time.  RoB: unclear sample frame |
| [94] | Hsieh et al.  (2015)  Taiwan | J Nurs Scholarsh | Quantitative: Cross-sectional, *correlational* | 187 Emergency Department nurses | Personality traits / **Social Network** **Integration** / Resilience | Resilience Scale (RS) / Social Support Scale (SSS) for family support and for peer support / Religious Beliefs: 1 item / Eysenck Personality Questionnaire (EPQ) | Higher degrees of peer support were associated with greater resilience among abused nurses. Among all forms of social network integration, only peer support was shown to enhance an individual’s resilience. |
| [95] | Escribà-Agüir & Perez-Hoyos  (2007)  Spain | Stress Health | Quantitative: Cross-sectional, *regression models* | 639 Emergency medical (Doctors (356) and nursing (279) staff | Mental health/ Vitality / Emotional exhaustion / Psychosocial work environment (psychological demands, job control, **supervisor social support and co-workers' social support**) | Health Survey (SF-36) (mental health, vitality) / MBI-HSS: EE  Job Content Questionnaire (JCQ) | Low co-workers’ social support at work was associated with poor psychological wel-being only among doctors. Low job supervisor’s social support increased the risk of high emotional exhaustion among nurses.  RoB: unclear sample frame |
| [96] | Bruyneel et al.  (2017)  Belgium | J Adv Nurs | Quantitative: Cross-sectional, *structural equation modeling* | 292 emergency nurses in 11 Emergency Departments | Turnover Intention / Work environment (**social support from supervisor / colleagues) /** Job Characteristics / Emotional Exhaustion | LQWQ-N / Nursing Work Index Practice Environment Scale (NWI-PES) / MBI-HSS: Emotional Exhaustion (EE) | Social support from supervisors had a significant effect on burnout of female nurses whom turnover intention was high. |
| ***Perioperative nurses*** | | | | | | | |
| [97] | Cronin-Stubbs & Brophy  (1985)  USA | J Psychosoc Nurs Ment Health Serv | Quantitative: Cross-sectional, *regression analysis* | 296 Nurses in psychiatric ward (66), OR (64), ICU (74), Medical specialty areas (91) | **Social support** (stability, availability, affirmation, affect, aid, size in personal or occupational **networks**) / Occupational stress (frequency, intensity) / Life Stress / Burnout | Norbeck Social Support Questionnaire (NSSQ) / Nursing Stress Scale (NSS) / Life Experience Survey (LES) / Staff Burnout Scale for Health / Professionals (SBS-HP) / Self developed Self-Report / Questionnaire (SRQ), semi-structured interviews | Operating room nurses experience more stress from interaction with physicians compared to other medical wards.  RoB: unclear sample frame |
| [102] | Chipps et al.  (2013)  USA | AORN J | Quantitative: Cross-sectional, *correlational* | 167 Perioperative personnel in 2 hospitals | **Bullying** / **Lateral violence** / Burnout | Negative Acts Questionnaire-revised (NAQ-R) / MBI | 59% of the participants reported witnessing coworker bullying weekly, and 34% reported at least two bullying acts weekly.  Significant differences between the two hospitals exited: one hospital three times more likely to be target of bullying than the other hospital.  Positive correlation of bullying with emotional exhaustion was found.  RoB: nonresponse bias, unclear sample frame |
| [103] | Dunn  (2003)  USA | AORN J | Quantitative: Cross-sectional, *correlational* | 145 Perioperative nurses | **Horizontal violence** / job satisfaction | Sabotage Savvy questionnaire / Index of Work Satisfaction (IWS) questionnaire | Although sabotage is reported as common in the OR, the presence of sabotage is not significantly associated with reported job satisfaction. |
| [104] | Park et al. (2015)  South Korea | J Nurs Scholarsh | Quantitative: Cross-sectional, *regression analysis* | 970 female nurses in 47 units (general, oncology, ICU, OR, Outpatient Department) | Work demands (quantitative, pace, emotional) / Trust and Justice / **Violence** (type and source) | Copenhagen Psychosocial Questionnaire (COPSOQ II) | Bullying was highest in operating rooms. Most frequent perpetrators of verbal abuse and sexual harassment were physicians. |
| [105] | Higgens & MacIntosh (2010)  Canada | Int Nurs Rev | Qualitative: Descriptive | 10 Operating room nurses | **Abuse surgeon-nurse** | Open-ended individual audiotaped interviews | Factors contributing to abuse were: culture in the OR, included environment and hierarchy, and resources and interpersonal relationships among physicians.  Nurses experienced negative psychological and social health effects from the abuse. |
| [106] | Timmons & Tanner  (2005)  UK | Int J Nur Pract | Qualitative: Inquiry | 17 theatre nurses, and 3 operating department practitioners (technicians) | **Relation Surgeon-nurse** –emotional work | Observation, interview | Emotional labor performed with coworkers was seen as necessary to maintain the ‘sentimental order’. |
| [107] | Gillespie et al.  (2007)  Australia | J Adv Nur | Quantitative: Cross-sectional*, regression models* | 772 nurses *(415 perioperative, 69 nurse anesthetists, 77 in combined role, rest OR personnel)* | Perceived competence / **Collaboration** / Control / Self-efficacy / Hope / Coping / Resilience | Perceived Competence Scale (PCS) / Collaboration with medical Staff (CMSS) / **Cohesion Among Nurses** (CANS) scales  **Peer Support scale** *(developed)*  Managing Stressful Situations scale (MSS) *(developed)* / General Self-Efficacy scale (GSE) Adult Dispositional Hope Scale (ADH) / Ways of Coping scale: Planful Problem-Solving (PSS) / CD-RISC | Collaboration was not a significant explanation of resilience in OR nurses.  RoB: unclear sample frame |
| [108] | Michael  (2002)  Australia | ACORN | Quantitative: Cross-sectional, *correlational*  Qualitative: *content analysis of narrations* | 213 Operating suite nurses | **Amount of available support / Sources** (co-worker, supervisors, organization)  Personal resources | Social resources questions following traumatic event descriptions / Sense Of Coherence (SOC) scale | Emotional support received through disclosure to colleagues and supervisors were the most salutary aspects.  Perioperative nurses reported low levels of support form nursing managers following traumatic events.  Coworkers dealt more effectively with the problems and the respondents’ feelings. |
| ***Nurse anesthetists*** | | | | | | | |
| [109] | Beuzekom et al. (2013)  Netherlands | Eur J Anaesthesiol | Quantitative: Cross-sectional, survey | 109 Specialist anesthetists, 46 trainee anesthetists, 115 nurse anesthetists | **Latent risk factors (LRFs)** / Job satisfaction / Job stress / Intension to leave | Leiden Operating Theatre and Intensive Care Safety (LOTICS) scale / Job LQWQ-Satisfaction scale / Modified stress assessment form. | Job satisfaction was most strongly related to teamwork and hierarchy. Hierarchy was strongly related to job stress. |
| [111] | Perry  (2006)  USA | AANA J | Qualitative: Inquiry | 20 CRNAs (+ 15 coworkers: anesthesiologists, RNs, technicians, surgeon) | Perceived occupational stress / Roles and responsibilities / Coping strategies | Semi-structured interviews (Nursing Stress Scale as a guide) /  Clinical observations / Artefact data | Interpersonal work relationships cause more stress than any of the other perceived job stressors. |
| [112] | Sakellaropoulos et al.  (2011)  USA | AANA J | Quantitative: cross-sectional, *descriptive*  Quantitative: Inquiry, content analysis | 205 CRNAs | Prevalence **workplace aggression** / Workplace stress | Workplace Aggression Research Questionnaire / 3 open-ended items | Workplace stress was significantly correlated with experiences of aggression (80%) by physician supervisors (58,4%) and coworkers (36,6%). |
| [113] | Elmblad et al. (2014)  USA | AANA J | Quantitative: Cross-sectional, survey | 385 CRNAs | **Incivility (bullying)** / burnout | Nursing Incivility Scale / Copenhagen Burnout Inventory / Qualitative data | A statistically significant direct relationship existed between workplace incivility and burnout.  The least prevalent source of incivility was supervisors. |
| ***Intensive Care Unit nurses*** | | | | | | | |
| [114] | Vermeir et al. (2018)  Belgium | Intensive Care & Critical Care Nursing | Quantitative: Cross-sectional, *descriptive, correlational* | 303 intensive care nurses (3 different hospitals) | **Communication** satisfaction / turnover intention/ Burnout/ Job satisfaction | Communication Satisfaction Questionnaire (CSQ)/ Questionnaire for the perception and Assessment of Labor: Turnover Intention Scale / Maslach Burnout Inventory (MBI) | Average job satisfaction was high.  Nurses were most satisfied about ‘communications with supervisors’. |
| [115] | Norbeck  (1985)  USA | Nursing Research | Quantitative: Cross-sectional, *regression models* | 164 female Critical Care nurses (8 hospitals; 18 critical care units) | **Sources of social support**  Perceived Social Support  Job Strain  Psychological symptoms | NSSQ / Questionnaire of Stressful Factors (32 items)/ Nursing Job Satisfaction Scale / SCL | Work support explained 24% of the variance of perceived job stress for married nurses. Support from relatives explained 10% of the variance in perceived job stress and 16% of the variance in psychological symptoms for unmarried group. |
| [116] | Klopper et al. (2012)  South Africa | Journal of Nursing Management | Quantitative: Cross-sectional, *correlational* | 935 Critical Care Nurses (62 hospitals) | **Practice environment**  Job satisfaction  Burnout | NWI-PES / MBI | The practice environment is positive, except for staffing and resource adequacy, and governance. CCN’s have a high degree of burnout. |
| [117] | Mohl et al. (1982)  USA | Psychosomatics: journal of Consultation and Liaison Psychology | Quantitative: Cross-sectional, descriptive | 68 Nurses in General Medicine Units (38) and ICUs (30) | Professional Self-Esteem (Ideal-image, Self-image) / Clinical Distress / Work attitudes and some **social system factors** | Osgood Semantic Differential / Hopkins Symptom Checklist (SCL-90) / **Work Environment** Scale (WES) | Social system variable contribute substantially to the stress level of ICU nurses. |
| [118] | Mrayyan  (2009)  Jordan | Contemporary Nurse | Quantitative: Correlational, *regression models* | 463 nurses: 228 from 12 ICUs and 235 from 9 medical wards (13 hospitals) | Job stress  **Social Support Behaviors** | Nursing Stress Scale (NSS) / Inventory of Social Support / Behaviours (ISSB) | Stressors in ICUs were higher than those in wards. The ICU’s scored higher than wards on the ‘conflict with physicians’ subscale of NSS. High job stress and low support behaviors were evidenced.  ICU nurses score higher on support behaviors toward other nurses than nurses working on medical wards. |
| [119] | Mahon  (2014)  Canada | Intensive Crit Care Nurs | Qualitative: Critical Ethnography | 31 Nurses in Pediatric Intensive Care Unit (PICU) | Work **experiences** / Job satisfaction / Retention Intention | Observation /Semi-structured interviews | The lack of autonomy and/or respect shown to nurses by administrators appears to be on of the major stressors in nurses’ working lives and can lead to attrition form the PICU. |
| [120] | McGibbon et al. (2010)  Canada | Qual Health Res | Qualitative: Institutional Ethnography, *thematic analysis* | PICU | Forms of stress in nurses / **Social organization of Nurses’ stress** (gender, hierarchies) | Tape-recorded In-depth interviews / Participant Observation / Focus groups / Field notes, research journal / Non-confidential texts | Six main forms of nurses’ stress, including emotional distress: negotiating hierarchical power. |
| [121] | Panunto & Guirardello (2013)  Brasil | Rev Lat Am Enfermagem | Quantitative: Cross-sectional, *structural equation modeling* | 129 nurses in 17 ICUs | Burnout  Perception of quality of care  Job Satisfaction  Intention to leave  Working environment **(nurse-physician relationship)** | NWI-R / MBI | Nurses with poor relationships with physicians experienced a greater level of emotional exhaustion. |
| ***Police officers*** | | | | | | | |
| [122] | Pelegrini et al. (2018)  Brazil | Cad. Bras. Ter. Ocup. | Quantitative: Cross-sectional, *regression models* | 84 civil & military male police officers | Perception of working conditions (**social environment: relationships with coworkers, supervisor**) / occupational stress | Environment and Working Conditions Profile questionnaire | As for occupational stress, more than half of the police identified their work as low demand, low control and low social supports. As for the perception of working conditions, she social environment was the component with the highest score.  RoB: No psychometric testing |
| [123] | Tuttle et al. (2018)  USA | The Family Journal: Counseling and Therapy for Couples and Families | Quantitative: Cross-sectional, *regression models* | 1.180 married law enforcement personnel | Work stress/ **Marital functioning** / Career demands / Social and emotional spillover family/ support | Police Officer Questionnaire | Career demands and emotional spillover were statistically significant predictors of the variance in martial functioning. Social and emotional spillover of work-related stress carries negative consequences for communications and emotion regulation within law enforcement marriages.  RoB: No psychometric testing |
|  | Brunetto et al. (2017)  Australia | Australian & New Zealand Journal of Criminology | Quantitative: Correlational, *factor analysis* | 193 police officers | Bullying (**intimidation, personal attacks**)/ Perceived organizational support from management/ psychological wellbeing/ affective commitment and turnover intentions | POS/ 14-items bullying (task attack, personal attack & intimidation) / (developed) psychological wellbeing (4-items) / Affective commitment to organization (6 items)/ turnover (3-items) | Perceived organizational support explained 6% of bullying. Perceived organizational support and bullying explained almost a third of psychological wellbeing. |
| [124] | Gachter et al. (2011)  USA | Policing: an international journal of Police Strategies & Management | Quantitative: Cross-sectional, *regression models* | 1100 Police officers | Psychological strain / Physical strain / Burnout Trauma / **Social capital at work** /  **Social capital at home** (‘stability at home’) | 5 different indices of stress *(constructed)* / narrow index social capital at work *(constructed)* / 9-item negative work-related event scale (constructed) / Index Stability at home *(constructed)* | An increase in social capital is significantly correlated to a decrease in the level of strain, in the psychological, physical, burnout and health areas.  Social capital in this work environment is by far the largest and most significant factor in reducing the negative effects of stress, closely followed by social capital at home.  RoB: no psychometric testing |
| [125] | Ojedokun & Balogun  (2015)  Nigeria | Span J Psychol | Quantitative: Cross-sectional, *structure equation modeling* | 340 Police personnel | Psychological Capital (hope, resilience, optimism, self-efficacy) / **Workplace Social Capital** / Mental Health | Psychological capital Questionnaire (24 items) / Social Capital Scale (8 items) / GHQ-28 | Vulnerability or coping with mental health outcomes was related to lower scores on psychological capital and workplace social capital.  Workplace social capital was shown to have an independent negative influence on somatic, anxiety, and depression symptoms. |
| [126] | Adams & Buck (2010)  USA | Crim Justice Behav | Quantitative: Cross-sectional, *descriptive analyses* | 196 police officers / employees (12 police departments) | External social stressors (civilians, suspects) / **Internal social stressors (coworkers, supervisors)** / Surface acting / Turnover intention / Psychological distress / Emotional exhaustion | Workplace Incivility Scale (7 items) / Customer Incivility Measure / 4 items from Emotional Labor Measure / 4 items the outcome of intent for turnover / 13 items psychological distress / 6 items Job-related EE | Social stressors from customers to strains also hold for social stressors arising from organizational insiders.  RoB: possible lack of psychometric testing |
| [127] | Duffy et al. (2002)  Republic of Slovenia | Academy of Management Journal | Quantitative: Cross-sectional, *hierarchical multiple regression* | 685 Police officers | **Social undermining** / **Enacted supervisor and coworkers supportive behaviors**/ Self-efficacy / Organizational commitment / Active and passive counterproductive work behaviors / Somatic complaints | Supervisor and Co-worker Undermining scales *(Developed and psychometric tested) /* Inventory of Social Supportive Behavior (ISSB) */* 4-item scale work-related self-efficacy */* 9-item Organizational Commitment Questionnaire*/* Items from 3 Counterproductive work behavior scales *(designed) /* 18 items Psychosomatic symptoms scale | Social undermining was significantly associated with employee outcomes, in most cases more strongly than was social support.  High levels of social undermining and support from the same source were associated with negative outcomes.  Support from one source appeared to only modestly attenuate the negative effects of social undermining from another source. |
| [128] | Beltrán et al. (2009)  Mexico | Span J Psychol | Quantitative: Cross-sectional, *descriptive and inferential analyses* | 875 traffic police agents | Burnout syndrome  **Received social support**: *objective / subjective*  Personal and Occupational Accomplishment | MBI-HSS / Diaz Veiga Social Resource Inventory / Personal and Occupational Accomplishment (FRP) | The social support networks designated as ‘low or poor’ were shown to be associated with burnout syndrome.  Strong networks of social support reported, were not strong enough effects to combat burnout syndrome. |
| [129] | Buunk & Verhoeven (1991)  Netherlands | Basic and applied social psychology | Quantitative: Cross-section study, during the course of a week | 40 Police officers | **Social interactions** at work / Number and nature of stressful events / Affect experienced at the end of the day / Organizational stress / Perceived social support  Health | Daily Interaction Record in Organizations (DIRO) / Organizational Stress Questionnaire (Van Dijkhuizen) | Perceived support by superiors was related more closely to features of social interaction than peer support by colleagues, whereas both cases the highest correlations were found with rewarding companionships.  The degree of intimate support provided by supervisors closely related to less negative stress at the end of the day. Intimate support by peers was linked with more reported negative affect. |
|  | ***Military personnel*** | | | | | |  |
| [130] | Luciano et al. (2017)  USA | The Journal of Nervous and Mental Disease | Quantitative: Cross-sectional, *correlational* | 63 OEF/OIF/OND veterans | PTSD / General health, resilience, social support, (post-deployment, **unit-social support**) | PCL-5 / RAND Short Form 36-Item Health Survey 1.0 (SF-36): 5 physical health subscales / **DRRI** | Military deployment social support moderated the relationship between PTSD and pain, whereas post-deployment social support moderated the relationship between PTSD and general health perceptions. |
| [131] | Milgram et al. (1989)  Israel | Military Psychology | Quantitative:  Cross-sectional, *correlational* | 48 combat veterans | Unit cohesiveness / Service-related stressors / Personal resources (goals, motivation) / Social support: **group cohesiveness** / military performance | Questionnaire 95 items: perceived exposure to personal danger, injuries and casualties others (6 items), Unpleasant emotional states (5 items), Unpleasant body sensations (9 items), Goals (2 items), Motivation (1 item), Group Cohesiveness (5 items), Primary loyalty (1 item), Group Morale (1 item), Satisfaction with functioning (1 item) | Soldiers reported no decline in unit cohesiveness.  Military performance was strongly associated with unit cohesiveness, and unrelated to intensity of combat-related stress. |
| [132] | Williams  (2016)  USA | Military Psychology | Quantitative: *longitudinal* (3-, 6-, and 9-week follow-up). | 1939 soldiers in Basic Combat Training, from 2 BCT battalions | Psychological distress / Sleep problems / Resilience / **Unit cohesion (group integration, personal bonding platoon)** / Confidence in managing personal reaction to stress / Basic combat training stressors / Positive state of mind / Performance measures | K6 / Sleep problems (4-item scale) / Resilience Scale (CD-RISC 2): 2 items (‘able to adapt to change’, ‘tend to bounce back after illness or hardship’)  Unit cohesion: 3-items from and 41-item scale (Podsakoff & MacKenzie) / Designed 11-items scale confidence in ability to manage cognitive, emotional and physical reaction / Positive state of mind scale (5-items) | Increased unit cohesion was associated with decreases in psychological stress reactions a positive state of mind. |
| [133] | Nevarez, Yee & Waldinger (2017)  USA | Journal of Traumatic Stress | Quantitative: cross-sectional, *hierarchical regression analysis* | 110 men with combat exposure (WWII) | **Relationship quality with fellow soldiers (in WWII) /** Quality early childhood relationships / emotional adjustment during college/ postwar PTSD symptoms | Document analysis multidisciplinary health service study / Combat Exposure Scale | Better peer relationship quality during deployment may reduce the likelihood of subsequent PTSD symptom development, and the quality of early relationships may set the stage for better relationships during stressful contexts such as ware.  RoB: no psychometric testing |
| [134] | Huang et al. (2017)  Taiwan | Suicide and Life-Threatening Behavior | Quantitative: Cross-sectional, *correlational* | 226 soldiers experiencing maladjustment and risk for suicide | **Thwarted belongingness** / perceived burdensomeness/ Suicide risk / alexithymia / personality / childhood trauma | 4-items based on Interpersonal-psychological theory / Toronto Alexithymia Scale/ EPQ / Mini-International Neuropsychiatric Interview / Brief Symptom Rating Scale (BSRS) | Loss of belongingness to the troop is a risk factor for suicidal tendencies. Maladjusted soldiers exhibit more thwarted belongingness and less fear of death than control participants. |
| [135] | Monteith et al. (2017)  USA | Journal of Clinical Psychology | Quantitative: cross-sectional, *hierarchical regression analysis* | 92 female veterans with history of military sexual trauma | Perceived burdensomeness/ **Thwarted belongingness** / fearlessness about death / suicidal ideation | INQ / Acquired Capability for Suicide Scale (ACSS-FAD) / Fearlessness about Death Scale / BSSI / BDI-II / PCL-C | When including all three interpersonal-psychological constructs in the model, only perceived burdensomeness and fearlessness about death were significantly associated with suicidal ideation. |
| [136] | Wesselman et al.  (2018)  USA | PLOS one | Quantitative: cross-sectional, *correlational* | 129 veterans (Army National Guard) | **Ostracism /** Psychological problems (posttraumatic stress symptoms, anxiety, psychological distress) / perceived social support (civilian and military source) | DRRI-2 / PCL-5 / State-Trait Anxiety Inventory: Anxiety trait subscale / Psychological need satisfaction measure (adapted) / 10 items perceived being ostracized | Veterans’ perceived ostracism (i.e., being ignored and excluded) correlated with psychological problems (i.e. posttraumatic stress symptoms, anxiety and psychological distress). |
| [137] | Kintzle et al. (2018)  USA | Healthcare | Quantitative: Cross-sectional, *correlational* | 722 veterans | **Social connectedness** / PTSD-symptoms / non-honorable discharge status / combat experiences | PCL-5 / Combat Experiences scale (CES)/ Social Connectedness Scale (8 items) | A positive direct effect was found for combat experiences and non-honorable discharge status on PTSD symptoms while social connectedness demonstrated a negative direct effect.  Both combat experiences and non-honorable discharge status demonstrated negative direct effects on social connectedness and indirect on PTSD through the social connectedness pathway. |
|  | Teo et al.  (2019)  USA | Journal of Affective Disorders | Quantitative: Cross-sectional, online survey | 587 military veterans | **Frequency of social contact** (in-person/ Facebook) / probable psychiatric disorders / suicidality | Health and Retirement Study and Pew Research survey (items adapted) / PC-PTSD / AUDIT-C / PHQ-2 / Depressive Symptom Inventory Suicidality Subscale (DSI-SS) | More frequent in-person social interaction was associated with significant decreased risk of symptoms of major depression and PTSD compared to contact every few weeks or less. |
| [138] | Bryan & Heron (2015)  USA | Depress Anxiety | Quantitative: Cross-sectional | 168 active duty Air Force convoy operators | **Belongingness** / depression/ posttraumatic stress | Patient Health Questionnaire / Depression Scale / PCL-Military  / INQ | Increased depression severity was significantly associated with low belongingness and with posttraumatic stress symptoms at 1, 3, 6 and 12 months following return. |
| [139] | Bryan et al. (2013)  USA | J Affect Disord | Quantitative: Cross-sectional, *regression models* | 273 active duty Air Force Security Forces Personnel | Suicidal ideation / Combat (aftermath) Exposure / Emotional Distress / **Belongingness** / Perceived burdensomeness | Beck Scale for Suicidal Ideation (BSSI) / Anxiety Depression Distress Index-27 (ADDI-27) / **DRRI** / INQ | A significant three-way interaction was found of age, combat exposure, and belongingness. Suicidal ideation was most sever among airmen above the age of 29 years with high Combat exposure and low levels of belongingness. |
| [141] | Ahronson et al. (2007)  Canada | Mil Psychol | Quantitative: Cross-sectional | 447 military employees (389 Army + 58 Navy) | **Group cohesion** / Job performance/ Job satisfaction / Psychological distress | Group Environment Questionnaire (GEO) / Job performance: 10 items / Job satisfaction: 31 items  Psychological distress: frequency general symptomatology 2 weeks | Dimensions of cohesion reflecting attraction to the group were inversely associated with psychological distress. |
| [142] | Kanesarajah et al. (2016)  Australia | Occupational Medicine | Quantitative: Cross-sectional, survey | 11411 military personnel deployed to Middle East, Iraq or Afghanistan | **Unit cohesion** / traumatic exposure / symptoms PTSD / psychological distress/ alcohol dependency | US Deployment Experiences Survey (5 questions unit cohesion) / DRRI (25 items traumatic exposure) / PCL-C / K10 / AUDIT | Those with low levels of unit cohesion had higher odds of PTSD symptoms, very high psychological distress and a high level of alcohol problems compared with those reporting high unit cohesion on deployment.  No significant interaction between unit cohesion and traumatic experiences was found in influencing poor mental health. |
| [144] | Brailey et al. (2007)  USA | J Traumatic Stress | Quantitative: Cross-sectional, *regression models* | 1.579 Army soldiers | **Unit cohesion** / PTSD symptoms / Live events | PCL / Deployment Risk and Resilience Inventory **(DRRI)** | Life experiences and unit cohesion strongly and independently predicted PTSD symptoms and unit cohesion attenuated the impact of life experiences on PTSD. |
| [145] | Breslau et al. (2016)  USA | J Affect Disord | Quantitative: Cross-sectional, *regression models* | 1.307 U.S. Marines before and after deployment to Iraq or Afghanistan | **Cohesion; unit-level,** individual level /  Mental health: alcohol misuse, violation of code (UCMJ), PTSD, depression | **DRRI** / Alcohol Use Disorders Identification-Consumption (AUDIT-C) / PCL-C /  Patient Health Questionnaire-2 (PHQ-2) / Patient Health Questionnaire-8 (PHQ-8) /Lifetime Events Checklist (LEC) | Unit-level cohesion is associated positively with alcohol misuse and violations but not with probable PTSD or a positive screen for depression. Lower perceptions of cohesion, relative to the other members of the same unit, are associated with higher likelihood of violations, probable PTSD and positive screen for depression. |
| [146] | Fontana et al. (1997)  USA | J Nerv Ment Dis | Quantitative: Cross-sectional, *regression models* | 1198 male theatre (Vietnam) veterans | **Unit cohesion / homecoming support** / PTSD/ psychopathology | Data from National Vietnam Veterans Readjustment Study (Kulka et al. 1990) | Unit cohesion had no significant relationship, as a direct effect, to either PTSD or other psychopathology. High levels op unit cohesion in combination with high war zone stress was associated with the highest levels of PTSD and psychopathology. Homecoming support was related negatively as a direct effect to both PTSD and other psychopathology. |
| [147] | Du Preez et al. (2012)  UK | Occupational Medicine | Quantitative: Cross-sectional, *regression models* | 4901 male Armed Forces personnel deployed to Iraq | **Unit cohesion** / Mental health (PTSD, common mental disorder, alcohol misuse) | Questions about: Childhood adversity / Relationship with family while deployed, **Unit cohesion (4 questions) /** PCL/ GHQ-12 / AUDIT | Perceived interest from seniors was associated with less probable PTSD and common mental disorder. Comradeship was associated with greater alcohol misuse. Feeling able to talk about personal problems was associated with less alcohol misuse among reserve personnel. |
| [149] | Smith et al. (2017)  USA | Anxiety, Stress, & Coping: An International Journal | Quantitative: Cross-sectional, *structural equation modelling* | 469 veterans who returned from deployment to Afghanistan or Iraq | Deployment-related experiences: warfare exposure, **sexual harassment,** concerns relationship disruption, deployment social support / Post-deployment Social Support / Current PTSD symptom severity | DRRI / Aftermath of Battle scales (15 items) / CES / Sexual Harassment scale (7 items) / Concerns about Life and Family Disruption scale / Deployment Social support (12 items)  **Post-deployment social support** Scale / PCL-M | Deployment factors predicted later PTSD symptoms though post-deployment. Social support and social reintegration, with lower support and higher social reintegration difficulty both associated with higher PTSD symptomatology. |
| [150] | Dutra et al. (2011)  USA | Journal of Trauma & Dissociation | Quantitative: Cross-sectional, *regression models* | 54 active duty women | Exposure combat experiences / **Military sexual harassment** / Post-deployment PTSD / Depressive symptoms | **DRRI** / Primary Care PTSD Screen (PC-PTSD) / CES-D: abbreviated version (2 items) | Combat experiences and sexual harassment jointly accounted for significant variance in post-deployment PTSD symptoms, whereas military sexual harassment was identified as the only unique significant predictor of these symptoms.  RoB: small sample size |
| [151] | Harris, McDonald & Sparks  (2018)  USA | Armed Forces & Society | Quantitative: cross-sectional, *correlational* | 21.304 active military duty members | **Organizational factors (e.g. climate, culture) /** | Defense Equality Opportunity Climate Survey (DEOCS) | Sexist environment context increases the likelihood of personal harassment experiences. Unit-level climate, group cohesion, and job satisfaction are not significant. The organizational context has less to do with culture or unit cohesion and more with tolerance of sexism. |
| [153] | Han et al. (2014)  USA | J Anxiety Disord | Quantitative: Cross-sectional, *regression models* | 835 Army soldiers and 173 national Guard Soldiers | **(Pre-deployment) Unit support / Post-deployment Social support** / PTSD symptoms | PCL-C / **DRRI** / Postdeployment Life Events Scale (17 items), Unit Support Scale, **Postdeployment Social Support** Scale (15 items) | Pre-deployment unit support was not significantly associated with post-deployment PTSD severity. Higher unit support during deployment was significantly associated with lower post-deployment PTSD severity among active duty soldiers. |
| [154] | Griffith  (2015)  USA | Behavioral Medine | Quantitative: Cross-sectional, *regression models* | 4.567 soldiers (of 50 company-sized units) | **Cross (unit)-level cohesion** / Suicide thoughts / Combat exposure/ Post-deployment Stressors / **Post-deployment Support / Unit cohesion** | US Army’s Reintegration Unit Risk Inventory (R-URI) (Social support: 3 items) | Post-deployment social support was associated with fewer suicidal thoughts. No evidence of the stress-buffering effect of social support was found. At group level, reduces risk for suicidal thought was associated with units having higher than average cohesion. |
| [155] | Boscarino et al. (2018)  USA | The Journal of Nervous and Mental Disease | Quantitative: Cross-sectional, *regression models* | 1730 deployed veterans (receiving outpatient care) | **Homecoming support** / Mental health (PTSD, depression, suicidal thoughts) | PTSD Checklist/ Depressive disorder scale based on DSM-4 diagnostic criteria/ BSI-18 / **DRRI**: Postdeployment section | Years after deployment, lower homecoming support was associated with current PTSD and suïcidality, regardless of theater and warzone exposure. For suïcidality, low social support had a greater impact on Iraq/Afghanistan veterans. |
| [156] | Hunt & Robbins (2001)  UK | Aging & Mental Health | Qualitative: Inquiry | 25 World War II (WWO-II) veterans from a sample (N=731) selected experienced potentially traumatizing events | **Social support (comrades / home)** / Coping strategies / Traumatic memories | Interviews *(identified different types of social support: comradeship, veterans’ association, wives and family)* | During war and 50 years after, comrades are valuable resource for discussing war experiences, and dealing with emotional content of traumatic recollections. Veterans tend not to discuss their traumatic memories with wives and families. |
| [157] | NayBack-Beebe & Yoder  (2011)  USA | Arch Psychiatr Nurs | Quantitative: cross-sectional, *correlational* | 137 Female service active duty members | **Social Support / Social Conflict** / Stressful life events / Mental health (depression, anxiety, PTSD) | Health and Social History instrument *(17 items, developed)* / **Interpersonal Relationships** Inventory-Short (26-item) (social support, social conflict) / DRRI /  PCL-M / Patient Health Questionnaire (PRIME-MD): Mood Module, Anxiety Module, Alcohol Abuse Module | Not the absence of social support but he presence of social conflict predicted increased  severity of PTSD symptoms and anxiety. |
| [158] | Sutker et al. (1995)  USA | J Abnorm Psychol | Quantitative: Cross-sectional, survey | 755 Persian Gulf War Returnees (Marine, Air Force, Navy, Army Reserve, National Guard), diverse functions | Perceived social support and satisfaction/ Family Support / **Family Cohesion** / prewar history/ PTSD diagnoses | Revised-SSQR / Family Relationship Index (FRI): *a measure of family relationships and support* / Family Environment Scale: subscale Cohesion | Environmental factors (cohesive family relationships) may alter vulnerability to negative ware stress outcomes.  PTSD diagnosis were associated with less family cohesion. |
| [159] | Solomon & Oppenheimer (1986)  Israel | Mil Med | Quantitative: Cross-sectional, survey | 99 Soldiers Yom-Kippur war + 100 control group soldiers | **5 social network variables**: Types of settlements (Kibbutz/Moshav), only child, bereaved family, soldier alone, immigrant. / Combat stress reactions | *Variables Immigrant, type of settlement and soldier alone reflect the* ***degree of integration*** *of the individual’ soldiers in their community.* | Soldiers coming from families hat appear vulnerable (less intense social network ties) were found to be more capable to withstand stress. |
| [160] | Karstoft et al. (2013)  Israel | J Clin Psychiatry | Quantitative: longitudinal,  *regression models* | male veterans with (369) and without (306) Combat stress reaction | PTSD / **Unit Support/** **Family environment / Social reintegration** | PTSD Inventory / Military Company Environment Inventory (emotional support, officer support, involvement in unit activities, order and coherence) /  Family Environment Scale (family cohesiveness, family expressiveness, family conflict)  7 questions based on Mueller’s social network interview about level of received support family and friends / Social Reintegration Scale | Reintegration difficulties after deployment are present in less than 20% of soldiers who return from Afghanistan. Difficulties are greater in individuals who follow symptomatic PTSD trajectories in the first years following deployment. |
| [161] | Solomon et al. (1991)  Israel | J Community Psychol | Quantitative: Cross-sectional, survey | Frontline soldiers (2 years after Lebanon war) 71 delayed PTSD and 73 PTSD + matched control group (73) | Life events / social resources (social integrations, loneliness, satisfaction with social network, **family environment**) / delayed PTSD | Life events: 36 item questionnaire  Social Reintegration Scale *(a feelings of alienation at homecoming, b belief that people and government support veterans)* / UCLA Loneliness Scale / Social network scale: *devised by authors* / Moos Family Environment Scale: **cohesiveness**, expressiveness and conflict | Delayed PTSD casualties evinced less social resources than did controls. |
| [162] | Hatch et al. (2013)  UK | Sociol Health & Illn | Quantitative: cross-sectional, *correlational* | 6.511 regular serving personnel (Armed Forces) and 1.753 regular service leavers | **Structural aspects of social integration** (Social Networks / Social participation outside work) / Mental health (Depression and Anxiety symptoms, PTSD-symptoms, Alcohol misuse) | GHQ-12 / PTSD checklist: 17 items / World Health Organization alcohol use disorder identification test (10-item) / Social participation: number of social organizations belong to or activities engaged in outside work.  Social Network Size (inside / outside military) | Service leavers reported less social participation outside work and a general disengagement with military social contacts in comparison to service personnel. The increased risk of common mental disorders but not PTSD symptoms was partially accounted for by reduced levels of social integration among service levers. Maintaining social network in which most members are still in the military is associated with alcohol misuse for both groups, but is related to CMD and PTSD symptoms for service leavers only. |
| [163] | Kaspersen et al. (2003)  Denmark | Scan J Psychol | Quantitative: Cross-sectional,  *regression models* | 72 UN Soldiers (army, navy, air force) + 141 relief workers | Trauma exposure / **Social Network Support** / PTSS | Trauma exposure: 8 questions /  Social Network Support: 7 questions / Post-traumatic Stress Scale (PTSS-10) / IES | All four network variables moderated the relationship between trauma exposure and post-trauma reactions among relief workers, while among UN soldiers only two such buffer effects were found.  Social support found to be important for UN soldiers low on trauma exposure. |
| [164] | Olson et al. (2018)  USA | Family Relations: Interdisciplinary Journal of Applied Family Science | Quantitative: cross-sectional, *hierarchical regression analysis* | 12,166 active duty Air Force personnel (in committed relationship) | PTSD / Stressful deployment experiences / Self-efficacy / Family coping / **Spouse/partner support /** Financial resources / **Neighbor support /** Religious participation / **Sense of community** | Air Force Community Assessment Survey / PC-PTSD / | Stressful deployment experiences were statistically related to elevated PTSD symptoms but also hat both personal and contextual factors moderated those symptoms. Spouse/ partner support moderated the relation between stressful deployment experiences and PTSD symptoms. |
| [165] | Thomas & Bowie  (2016)  USA | Journal of Social Service Research | Quantitative: Cross-sectional, survey | 131 military **veterans** attending a vet fair in efforts to find employment | **Sense of Community** / mental health (PTSD, depression, suicidal ideation), employment status / Veteran Community reintegration difficulties (interpersonal relationships with family, friends, peers. Productivity at work, school or home. Community participation. Self-care. Leisure and Perceived meaning in life. | Brief Sense of Community Scale (BSCS) / 4-item PC-PTSD /  CES-D 10 / SBQ-R *(1 item of this scale /* / Ultra-Short-Form Suicidal Ideation Thinking Measure (USSTM) *(1 item of this scale)* / Military to Civilian Questionnaire (M2CQ) | Veterans who are connected to their local communities may be more at risk for depression and community reintegration difficulties. |
| [166] | Molendijk (2018)  Netherlands | Social Science & Medicine | Qualitative: grounded theory | 20 Bosnia veterans, 20 Afghanistan veterans | Moral injury / Dutch national attitudes towards the military | Interviews | Public criticism and admiration may both be experienced as misrecognition, and, in turn, societal misrecognition may directly or indirectly contribute to moral injury. |
| [167] | Cederbaum et al. (2017)  USA | Public Health Reports | Process modeling mediation analysis | 321 military personnel (partnered) | **Social support** / mental health (depression, PTSD, Anxiety) / dyadic functioning | Multidimensional Scale of Perceived Social Support plus 2 sources of support (peers and leaders) / Patient Health Questionnaire-9 / PCL-M/ Generalized Anxiety Disorder scale / Dyadic Adjustment Scale | Dyadic functioning mediated the relationship between social support and depression/PTS only when social support came from nonmilitary friends or family. Dyadic functioning mediated social support and anxiety only when support came from family. No indirect effects of support from military peers or military leaders. |
